# ‘Caveat emptor’: the cautionary tale of endocarditis and the potential pitfalls of clinical coding data—an electronic health records study

**DOI:** 10.1186/s12916-019-1390-x

**Published:** 2019-09-04

**Authors:** Nicola Fawcett, Bernadette Young, Leon Peto, T. Phuong Quan, Richard Gillott, Jianhua Wu, Chris Middlemass, Sheila Weston, Derrick W. Crook, Tim E. A. Peto, Berit Muller-Pebody, Alan P. Johnson, A. Sarah Walker, Jonathan A. T. Sandoe

**Affiliations:** 1 National Institute for Health Research (NIHR) Health Protection Research Unit on Healthcare Associated Infections and Antimicrobial Resistance, John Radcliffe Hospital, Headley Way, Oxford, OX3 9DU UK; 2 Nuffield Department of Medicine, University of Oxford, John Radcliffe Hospital, Headley Way, Oxford, OX3 9DU UK; 3 Oxford University Hospitals NHS Foundation Trust, John Radcliffe Hospital, Headley Way, Oxford, OX3 9DU UK; 4 NIHR Biomedical Research Centre, Oxford, OX3 9DU UK; 5 Department of Cardiology, Leeds Teaching Hospitals NHS Trust and University of Leeds, Leeds, LS1 3EX UK; 6 School of Dentistry, University of Leeds, Leeds, LS2 9LU UK; 7 National Infection Service, Public Health England, Colindale, London, UK; 8 Department of Microbiology, Leeds Teaching Hospitals NHS Trust and University of Leeds, Leeds, LS1 3EX UK; 9 Microbiology Level 7, John Radcliffe Hospital, Headley Way, Oxford, OX3 9DU UK

**Keywords:** Electronic health records, Coding, Big data, Endocarditis, ICD-10

## Abstract

**Background:**

Diagnostic codes from electronic health records are widely used to assess patterns of disease. Infective endocarditis is an uncommon but serious infection, with objective diagnostic criteria. Electronic health records have been used to explore the impact of changing guidance on antibiotic prophylaxis for dental procedures on incidence, but limited data on the accuracy of the diagnostic codes exists. Endocarditis was used as a clinically relevant case study to investigate the relationship between clinical cases and diagnostic codes, to understand discrepancies and to improve design of future studies.

**Methods:**

Electronic health record data from two UK tertiary care centres were linked with data from a prospectively collected clinical endocarditis service database (Leeds Teaching Hospital) or retrospective clinical audit and microbiology laboratory blood culture results (Oxford University Hospitals Trust). The relationship between diagnostic codes for endocarditis and confirmed clinical cases according to the objective Duke criteria was assessed, and impact on estimations of disease incidence and trends.

**Results:**

In Leeds 2006–2016, 738/1681(44%) admissions containing any endocarditis code represented a definite/possible case, whilst 263/1001(24%) definite/possible endocarditis cases had no endocarditis code assigned. In Oxford 2010–2016, 307/552(56%) reviewed endocarditis-coded admissions represented a clinical case. Diagnostic codes used by most endocarditis studies had good positive predictive value (PPV) but low sensitivity (e.g. I33-primary 82% and 43% respectively); one (I38-secondary) had PPV under 6%. Estimating endocarditis incidence using raw admission data overestimated incidence trends twofold. Removing records with non-specific codes, very short stays and readmissions improved predictive ability. Estimating incidence of streptococcal endocarditis using secondary codes also overestimated increases in incidence over time. Reasons for discrepancies included changes in coding behaviour over time, and coding guidance allowing assignment of a code mentioning ‘endocarditis’ where endocarditis was never mentioned in the clinical notes.

**Conclusions:**

Commonly used diagnostic codes in studies of endocarditis had good predictive ability. Other apparently plausible codes were poorly predictive. Use of diagnostic codes without examining sensitivity and predictive ability can give inaccurate estimations of incidence and trends. Similar considerations may apply to other diseases. Health record studies require validation of diagnostic codes and careful data curation to minimise risk of serious errors.

**Electronic supplementary material:**

The online version of this article (10.1186/s12916-019-1390-x) contains supplementary material, which is available to authorized users.

## Background

Electronic health records are a powerful resource, enabling large observational analyses to be undertaken to assess disease outcomes, monitor trends and assess the effectiveness of healthcare. Their routine collection means that their use in research does not place an additional data collection burden on National Health Service (NHS) staff. Identification of diseases in health records is frequently based on analysis of the World Health Organization ICD-10 [1] diagnostic codes assigned to a patient’s hospital admission. Whilst the process of recording these codes upon discharge is internationally standardised and audited, these codes are recorded principally for reimbursement and administration, and multiple sources of potential error exist in the process of assigning codes [2, 3]. Previous studies have shown how coded data can create artefactual patterns in mortality [4].

Endocarditis is a useful and clinically relevant ‘test case’ for studying electronic health record accuracy. It benefits from having objective clinical criteria for defining true diagnoses and shares little overlap with other conditions. Additionally, the low overall incidence of infective endocarditis, even in high-risk populations, means that very large-scale and resource-intensive individually randomised controlled trials would be required to test the benefits of preventative interventions. Thus, electronic health record studies have been particularly important in guiding the management of infective endocarditis.

Numerous studies have been performed worldwide to assess the impact of changes to recommendations on the use of antibiotic prophylaxis to prevent infective endocarditis [5–10], with a lack of clear consensus [11–23] (summary in Additional file 1: Table S1 and Table S2). Some studies have found no significant change in disease trends after guidelines stopped recommending routine antibiotic prophylaxis for a broad range of at-risk individuals. Other studies suggest that any increase in overall incidence may be driven by an increasing population of ‘at-risk’ older adults, including individuals with predisposing heart conditions and prosthetic devices [19]. The largest studies suggesting an increase in endocarditis incidence after guidelines changed used US health insurance data [24], and English Hospital Episode Statistics (HES) data [14]. Given the lack of randomised controlled trials, these studies form some of the best available evidence; it is therefore important that the validity and accuracy of coding data, which these studies use, are comprehensively assessed.

The largest study investigating accuracy of endocarditis coding considered 1673 hospitalisations at a US centre and found that sensitivity for identifying true infective endocarditis cases ranged from 21.1 to 97.2% depending on the definition of endocarditis, and diagnostic codes included [19]. In contrast, endocarditis coding quality in England has not been explored in detail to date; this is particularly relevant because an English study suggested increasing incidence after changes in dental prophylaxis [14]. Given the importance of electronic health record data in endocarditis, and the utility of endocarditis as a case study given differences in coding algorithms in previous studies (Additional file 1: Table S2), we investigated the quality of endocarditis diagnostic coding data in two English tertiary care centres, combining retrospective audit, service evaluation, linked electronic health record and microbiology data. Admissions with an endocarditis diagnostic code were compared with recorded cases of infective endocarditis based on objective criteria, incidence trends in coded and confirmed clinical cases were assessed and reasons for discrepancies were explored.

## Methods

### Study population

Coding was studied in Leeds Teaching Hospital NHS Trust (Leeds), comprising seven tertiary and secondary care centres, directly serving a population of 780,000 with 1785 beds, and in Oxford University Hospitals NHS Foundation Trust (Oxford), a teaching hospital with three associated tertiary care centres, serving a population of 655,000 with 1465 beds [25].

### Identifying diagnostic codes for infective endocarditis and secondary organisms

We reviewed all diagnostic codes from the WHO ICD-10 Version 5 containing the word ‘endocarditis’, together with codes used in previous publications related to infective endocarditis and causative organisms in electronic health records (including ICD-10 equivalents of ICD-9 codes) [11, 13, 17–20, 22, 26, 27], and codes used for confirmed clinical endocarditis cases in a 2016 audit of Oxford data. These were reviewed by three clinicians (NJF, BY, LP) and the Oxford clinical coding team (CM, SW) and classified as ‘included in study’ (represents infective endocarditis of non-viral aetiology) or ‘not included’ (represents disease entity other than as defined by standardised criteria), or ‘not present in UK data’ (Table 1). Supplementary codes representing specific organisms were similarly reviewed and classified as representing the most common causative pathogens, *Streptococcus* spp., *Staphylococcus* spp. or others (Additional file 1: Table S3).

**Table 1:** ICD-10 Endocarditis codes and corresponding ICD-9 codes (and clinical modifications)

ICD-10 Code	Description	Corresponding ICD-9 Code/ICD-9-CM code	Description
Included			
I33 (I330)	Acute and subacute infective endocarditis	4210	Acute and subacute infective endocarditis
I38	Endocarditis, valve unspecified	4249	Endocarditis valve unspecified cause
(I38.X)		42499	Other endocarditis valve unspecified
I339	Acute and subacute endocarditis, unspecified	4219	Acute endocarditis unspecified
T826	Infection and inflammatory reaction due to cardiac valve prosthesis	99661	Infection and inflammatory reaction due to cardiac device implant and graft
B376	Candidal endocarditis	11281	Candidal endocarditis
I39	Endocarditis and heart valve disorders in diseases classified elsewhere	11504	*Histoplasma capsulatum* endocarditis
(I390)			Histoplasma duboisii endocarditis
		11514	Histoplasmosis endocarditis
		11594	Acute and subacute infective endocarditis in diseases classified elsewhere
		4211	Endocarditis in diseases classified elsewhere
I398	Endocarditis, valve unspecified, in diseases classified elsewhere	42491	
Not included			
I423	Endomyocardial (eosinophilic) disease	4250	Endomyocardial fibrosis
I091	Rheumatic diseases of the endocardium, valve unspecified	3979	Rheumatic diseases of the endocardium valve unspecified
T827*	Infection and inflammatory reaction due to other cardiac and vascular device, implants and grafts	99661	Infection and inflammatory reaction due to cardiac device implant and graft
		99662	Infection and inflammatory reaction due to vascular device implant and graft
I011	Acute rheumatic endocarditis	3911	Acute rheumatic endocarditis
Codes that feature only US specialised coding guidance (ICD-10-CM 2010) [27], not in UK datasets			
A3951	Meningococcal endocarditis	3642	Meningococcal endocarditis
A3282	Listerial endocarditis	42491	Endocarditis in diseases classified elsewhere
A5203	Syphilitic endocarditis	42491	Endocarditis in diseases classified elsewhere
B3321	Viral endocarditis	7422	Coxsackie endocarditis
A5483	Gonococcal heart infection	9884	Gonococcal endocarditis

*The T827 code (‘Infection and inflammatory reaction due to other cardiac and vascular device, implants and grafts’) was not selected as a review of the Oxford 2016 audit data suggested it was overwhelmingly used for wound infections after surgery, and the number of admissions with a primary or secondary T827 code was greater than all other endocarditis-coded admissions combined

### Data sources

#### Clinical cases of infective endocarditis: endocarditis service database (Leeds) and clinical audit (Oxford)

In Leeds, patients with suspected infective endocarditis referred by physicians across all sites have been reviewed by a dedicated team prospectively since 1 January 2006 and clinical details recorded in the Leeds Endocarditis Service Database, including modified Duke criteria [28, 29] (definite, possible, rejected [i.e. investigated and excluded]), causative organism genus, local patient identifier and admission dates (Additional file 1: Figure S1). Electronic notes for admissions 2006–2016 with an endocarditis code but no corresponding record in the endocarditis service database were also reviewed retrospectively as part of a service evaluation exercise.

In Oxford, electronic and paper notes from endocarditis-coded admissions 2010–2016 were retrospectively reviewed in an audit of endocarditis coding (Additional file 1: Extended Methods).

As this did not provide information on endocarditis cases which did not receive an endocarditis diagnostic code, we additionally reviewed notes from all patients who had antibiotics prescribed for infective endocarditis in Jan-Dec 2016, within a service evaluation of antibiotic prescribing (Additional file 1: Figure S3). Data prior to 2016 were not available as electronic prescribing was only implemented in late 2015.

#### Electronic health record data

In Leeds, electronic health record data were extracted from hospital databases as part of a service evaluation exercise for all admissions of patients in the Leeds Endocarditis Service Database, and all admissions with an endocarditis diagnostic code 2006–2016 inclusive. In Oxford, electronic health record data were extracted from hospital databases for all admissions during 2010–2016 with an endocarditis code, and for 2016 for admissions with a prescription indicating endocarditis. Data were extracted separately from an anonymised linked data warehouse [30] for all admissions with an endocarditis code from 1999 to 2016 for epidemiological analyses.

#### Microbiological data on causative organisms

For Leeds, organisms causing endocarditis were recorded by the clinician at diagnosis, based on microbiology results from a fully accredited UK microbiology laboratory, which followed standardised procedures in bacterial culture, identification and susceptibility testing [31–33]. For the Oxford 2010–2016 audit, causative organism was based on the organism recorded in the medical notes. For the Oxford 1999–2016 epidemiological analysis, causative organism was the organism isolated from blood culture (or *Bartonella/Coxsiella* serological testing) taken closest to the date of admission and during the admission, or up to 7 days preceding the admission if no organism was isolated during the admission. Organism identification was from a similarly accredited UK microbiology laboratory.

#### Variables

Anonymised electronic health record data extracted in Oxford and Leeds included admission/discharge dates, method of admission/discharge and all diagnostic codes from all consultant episodes. In Oxford, data on blood cultures and *Bartonella/Coxsiella* serological testing as above were included from the anonymised linked data warehouse [30].

### Data processing

#### Defining endocarditis cases

All cases (Leeds) and admissions (Oxford audit) evaluated as fulfilling modified Duke criteria [28, 29] for possible or definite endocarditis were included in the analysis. Briefly, this guidance identifies major criteria (such as repeated blood cultures positive for typical microorganisms and echocardiographic demonstration of valvular involvement) and minor criteria (such as fever, predisposing factors, limited microbiological evidence and other systemic features). Definite cases fulfilled 2 major criteria, 1 major criterion and 3 minor criteria or 5 minor criteria. Possible cases fulfilled 1 major criterion and 1 or 2 minor criteria, or 3 minor criteria.

#### Classifying admissions in electronic health record data

An admission was defined as a hospital provider spell (‘the total continuous stay of a patient [..] on premises controlled by a Health Care Provider’) according to NHS Business definitions [34]. Each spell comprised a number of consultant episodes, each with a primary ICD-10 code (the main condition treated or investigated) and up to 20 secondary codes for other relevant conditions and/or supplementary codes, e.g. reflecting organisms isolated (subsequently denoted ‘secondary codes’). An admission with an endocarditis code was defined as any spell where an infective endocarditis code was used in any position of any consultant episode. If more than one endocarditis code was used during the spell, the primary code(s) was prioritised followed by secondary codes, with code priority being I33.0>I33.9>I39.0>139.8>I01.1>I09.1>I42.3>B37.6>T82.6>I38.0 based on a priori clinical plausibility and use in previous studies (Table 1 and Additional file 1: Table S1). For admissions matched to an infective endocarditis case with no associated endocarditis code, we chose the dominant episode using previously reported methods to assess the coded reason for admission [35].

#### Data matching

All cases of infective endocarditis identified in the Leeds Service Database or Oxford 2016 prescribing evaluation were matched to admissions in electronic health record data, based on local patient identifier and nearest admission/discharge dates. In cases of multiple matches, admissions with an endocarditis code, followed by the longest admission during the clinician-recorded endocarditis dates, were chosen. 9/1541 (0.006%) patients reviewed in Leeds could not be matched to any inpatient admission and were not considered further (Additional file 1: Figure S1). JS and RG had full access to the Leeds Endocarditis Service Database and admissions with endocarditis codes in Leeds. NF had access to an anonymised extract of the Leeds Endocarditis Service Database and linked admissions with endocarditis codes. NF had full access to the anonymised database of admissions to Oxford with an endocarditis code and linked blood culture results. NF also had full access to the audit database of admissions to Oxford with an endocarditis code and the audit prescription database. However, no author had access to the underlying population of all admissions to the two hospitals.

#### Classifying readmissions

In the analysis of coding data vs confirmed clinical cases, admissions that did not directly match a case of endocarditis were classified as readmissions for infective endocarditis if the admission occurred within 30 days of a discharge date from a spell with an endocarditis code. If the patient had a previous diagnosis (determined by the clinician) or previous admission with an endocarditis code > 30 days previously, this was counted as a past history. Length of stay was calculated as calendar date of discharge minus date of admission.

#### Improving case identification using administrative data

To improve identification of confirmed clinical cases from electronic health records, based on the findings of the comparisons of endocarditis-coded vs confirmed clinical cases, we examined the utility of excluding short stays, apparent readmissions and elective admissions. Based on clinical experience, it was judged unlikely that a patient with infective endocarditis would be admitted and discharged alive in less than 5 days. In the Oxford 2010–2016 audit, there were no admissions < 3 days surviving to discharge that represented a case. In Leeds, 373 endocarditis-coded admissions < 3 days survived to discharge; only 3 (1%) were confirmed clinical cases. We therefore considered a threshold of < 3 days (discharge date minus admission date) to exclude implausible endocarditis-coded admissions.

A normal treatment plan for endocarditis would be at least 6 weeks’ antibiotics. In the Oxford 2010–2016 audit, two admissions of < 6 weeks were confirmed clinical cases—both patients needed emergency valve surgery for the initial case of endocarditis, then developed endocarditis of the new valve with different organisms within 6 weeks, but after 30 days. As our aim was to investigate thresholds that minimised loss of true cases (and prioritised preserving sensitivity), we considered a threshold of < 30 days from the previous discharge date to exclude readmissions.

Elective admissions were defined as admission method 11 (waiting list), 12 (booked) or 13 (planned) [34]. In Oxford, 33 elective admissions with an endocarditis code were identified; all were true elective admissions and 10 represented confirmed clinical cases, being elective admissions for valvular surgery and postoperative endocarditis (5, 3 and 2 were admission methods 11, 12 and 13 respectively).

#### Identifying confirmed clinical cases from prescribing data in Oxford

We searched for endocarditis cases using the mandatory ‘indication’ field which all clinicians have to complete to prescribe an antibiotic on the electronic prescribing system. We manually inspected the records of all patients with a prescription January–December 2016 with indication matching text string ‘ndoca’, ‘ie’, ‘valve’, ‘aortic root’ and ‘vegetation’ (fuzzy text search) (Additional file 1: Figure S3).

#### Statistical methods

Analyses were performed using STATA 13.1. Incidence trends were estimated from annual counts using Poisson regression as there was no evidence of overdispersion (*p* > 0.4), using population data for Oxfordshire and the Leeds area from the Office of National Statistics [36] for each year from 2001 to 2016 as an offset (imputing 2001 data for 1999 and 2000 in Oxford).

## Results

### Less than half of admissions with an endocarditis code recorded in electronic health records represented a confirmed clinical case of infective endocarditis, driven mostly by the I38 (endocarditis: valve unspecified) code

**Fig. 1:**
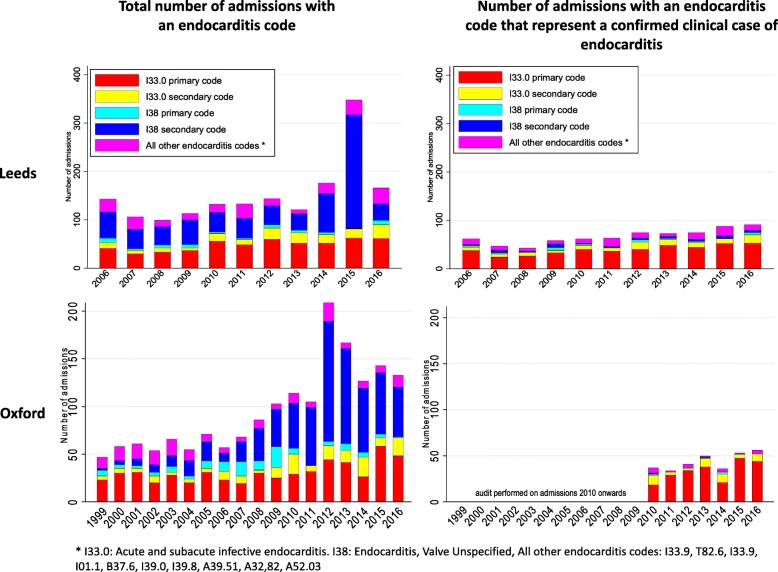
Numbers of admissions with endocarditis codes in Leeds and Oxford, compared to those of admissions that represent a new clinical case

**Fig. 2:**
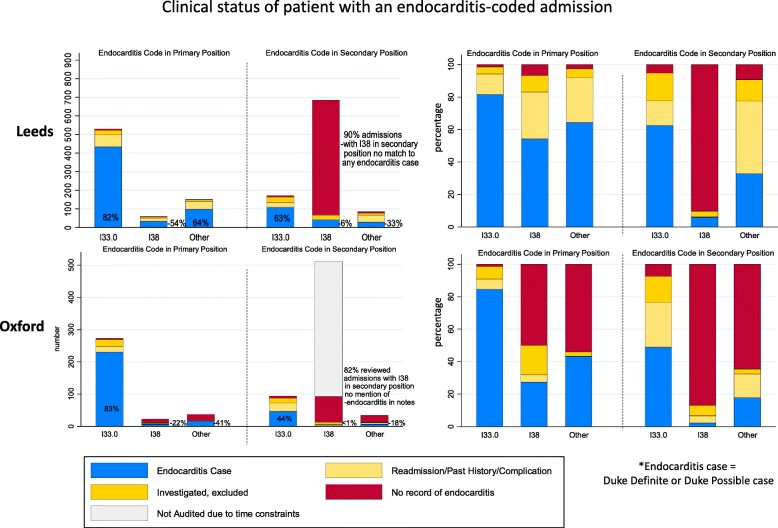
Clinical status of patients with endocarditis-coded admissions in Leeds and Oxford

1681 and 1725 admissions with an endocarditis diagnostic code in the primary or secondary position were identified in Leeds (2006–2016) and Oxford (1999–2016), respectively (Fig. 1, Additional file 1: Figure S1 and Figure S2). In Leeds, 738/1681 (44%) endocarditis-coded admissions between 2006 and 2016 represented Duke definite/possible cases (Fig. 2 and Table 2). In Oxford, 307/552 (56%) reviewed admissions between 2010 and 2016 represented Duke definite/possible cases (Figs. 1 and 2).

**Table 2:** Leeds data: Agreement between admissions and cases with coding combinations with short admissions, readmission and elective admissions removed

Codes used	Removal of admissions with:										
	< 3 day LOS	Readmissions < 30 days	Elective status	True positive	False positive	True negative	False negative	Sensitivity	Specificity	PPV	NPV
I33.0 primary only				433	97	1664	568	0.43	0.95	0.82	0.75
I33.0 primary only	✓			431	67	1694	570	0.43	0.96	0.87	0.75
I33.0 primary only	✓	✓		426	65	1696	575	0.43	0.96	0.87	0.75
I33.0 primary only	✓	✓	✓	406	57	1704	595	0.41	0.97	0.88	0.74
All primary codes				562	178	1583	439	0.56	0.90	0.76	0.78
All primary codes	✓			559	114	1647	442	0.56	0.94	0.83	0.79
All primary codes	✓	✓		552	111	1650	449	0.55	0.94	0.83	0.79
All primary codes	✓	✓	✓	529	96	1665	472	0.53	0.95	0.85	0.78
I33.0 in any position				549	162	1599	452	0.55	0.91	0.77	0.78
I33.0 in any position	✓			547	125	1636	454	0.55	0.93	0.81	0.78
I33.0 in any position	✓	✓		540	117	1644	461	0.54	0.93	0.82	0.78
I33.0 in any position	✓	✓	✓	514	102	1659	487	0.51	0.94	0.83	0.77
All primary and I33.0 secondary				669	242	1519	332	0.67	0.86	0.73	0.82
All primary and I33.0 secondary	✓			666	172	1589	335	0.67	0.90	0.80	0.83
All primary and I33.0 secondary	✓	✓		658	163	1598	343	0.66	0.91	0.80	0.82
All primary and I33.0 secondary	✓	✓	✓	629	141	1620	372	0.63	0.92	0.82	0.81
All codes except I38 secondary				697	299	1462	304	0.70	0.83	0.70	0.83
All codes except I38 secondary	✓			694	209	1552	307	0.69	0.88	0.77	0.84
All codes except I38 secondary	✓	✓		686	196	1565	315	*0.69*	*0.89*	*0.78*	*0.83*
All codes except I38 secondary	✓	✓	✓	653	168	1593	348	0.65	0.91	0.80	0.82
All codes				738	943	818	263	0.74	0.47	0.44	0.76
All codes	✓			735	573	1188	266	0.73	0.68	0.56	0.82
All codes	✓	✓		726	538	1223	275	0.73	0.69	0.57	0.82
All codes	✓	✓	✓	693	487	1274	308	0.69	0.72	0.59	0.81

Note: Total number of clinically confirmed endocarditis cases = true positives + false negatives (1001); total number of endocarditis admissions = sum of all four columns (2762)

### Some codes used in most endocarditis studies had good predictive ability, but the frequently used I38 code represented a confirmed clinical case in < 6% of admissions

Not all diagnostic codes were equal—the code I33.0 (‘Acute and subacute infective endocarditis’) in the primary position (‘the main condition treated or investigated during the relevant episode of healthcare’ [1]), included in most endocarditis studies (Additional file 1: Table S2), represented a new case in 433/530 (positive predictive value (PPV) 82%) and 231/273 (PPV 85%) reviewed admissions in Leeds and Oxford, respectively (Fig. 2). Non-I33.0 codes and those in secondary positions performed less well, but some rarer codes nevertheless represented true cases, particularly in the primary position. Among endocarditis secondary codes (‘all conditions that co-exist at the time of admission, that develop subsequently, or that affect the treatment received and/or the length of stay’ [1]), the code I38 (‘Endocarditis, valve unspecified’) was the most commonly used, but represented a new case in only 41/685 (PPV 6%) and 2/97 (PPV 2%) reviewed admissions in Leeds and Oxford, respectively (Fig. 2); 619 (90%) and 80 (82%) respectively had no mention of endocarditis in their medical notes, though many had some form of valvular heart disease. Both centres showed an apparent increase in the number of endocarditis-coded admissions over time, with sudden spikes at different time points (2015 in Leeds, 2012 in Oxford), driven largely by admissions with a secondary I38 code (Fig. 1).

### Discrepancies between codes and confirmed clinical cases were mainly due to correctly assigned codes for readmissions, past histories and investigations for endocarditis (later excluded)

The majority of admissions with an endocarditis code that were not confirmed clinical cases had legitimate reasons for the code being assigned. A readmission or relevant past history accounted for 190/1681 (11%) and 53/552 (10%) endocarditis-coded reviewed admissions in Leeds and Oxford, respectively (Fig. 2). Admissions where infective endocarditis was investigated and ruled out accounted for 101/1681 (6%) and 48/552 (9%) admissions in Leeds and Oxford, respectively. Discussions with the Oxford clinical coding team confirmed the NHS Clinical Classifications Service guidance [37] that a patient referred by a General Practitioner for ‘suspected endocarditis’, who had the diagnosis later excluded with no other definitive diagnosis confirmed would be correctly assigned a primary I33.0 code.

### I38: ‘Endocarditis: valve unspecified’ could be correctly assigned even if endocarditis was never mentioned in the notes due to indexing guidance

Review of the coding process identified that the WHO ICD-10 Alphabetical index directs many non-specific conditions towards an endocarditis code. For instance, ‘Stenosis-> valve (cardiac)(heart) (see also Endocarditis) I38’. This was discussed with the Clinical Classifications Service, UK, the definitive source of clinical coding guidance who set the national standards for ICD-10 used by the NHS, who responded: ‘a coder would be correct to assign code I38 when indexing a documented diagnosis which leads the coder to assign code I38, even when the term endocarditis is not documented within the medical record’. (Full quote in Additional file 1: Extended Methods.)

### Secondary codes often represent Duke definite/possible cases; primary codes miss a quarter of these cases

Patients who presented with embolic phenomena (e.g. stroke or cerebral abscess) due to infective endocarditis, or who developed infective endocarditis during an admission for valve surgery or chemotherapy, were commonly assigned a secondary endocarditis code, and a primary code reflecting the presentation, following coding guidelines. In Leeds and Oxford, 176/738 (24%) and 54/307 (25%) definite/possible cases with an endocarditis diagnostic code had this as a secondary code, respectively (Additional file 1: Figure S1 and Figure S2).

### A quarter of Duke definite/possible endocarditis cases may not receive any endocarditis diagnostic code and are not readily identifiable using electronic health records

In Leeds, there were 1001 Duke definite/possible cases during 2006–2016 (Additional file 1: Table S4), of which 263 (24%) did not have an endocarditis diagnostic code associated with their admission (sensitivity 76%). This occurred less commonly for Duke definite (153/713 (21%)) versus Duke possible (110/288 (38%)) cases (*p* < 0.0001). Fifty-two (20%) missed cases had the code ‘T82.7: Infection and inflammatory reaction due to other cardiac and vascular devices, implants and grafts’ present (primary/secondary), but other primary codes covered a diverse range of infection, sepsis and heart disease codes with no clear pattern (Additional file 1: Figure S1).

In Oxford, an audit of 2016 electronic prescribing records identified 10 additional cases above the 66 identified by diagnostic codes (Additional file 1: Figure S3) (sensitivity 87%). Five had pacemaker lead infections with a code indicating an infected device, two were cancer patients developing infective endocarditis as inpatients, one had coding reflecting a septic, ischaemic foot and intensive care management with endocarditis found during the admission, one aortic root abscess had ‘arteritis’ written on a discharge summary and was coded as such and one had coding for a mitral valve disorder with streptococcal sepsis.

### Raw endocarditis-coded admission data can give inflated estimations of incidence which can be mitigated by curation using carefully selected codes and other administrative data

**Fig. 3:**
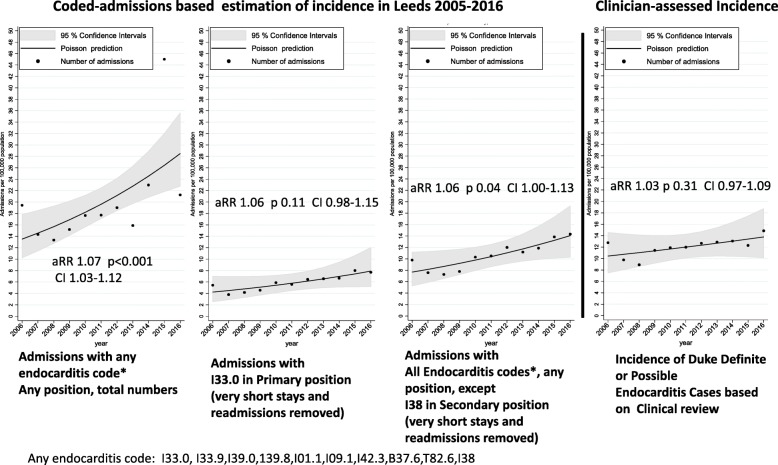
Incidence of endocarditis in Leeds as estimated by electronic health records, compared to the clinical case

Estimating cases of infective endocarditis using all admissions and all endocarditis codes (as defined in Table 1) overestimated the apparent incidence in Leeds during 2006–2016 by over twofold compared to confirmed clinical cases in the Leeds service database (sensitivity/specificity/positive predictive value [PPV] 0.74/0.47/0.44 respectively) (Fig. 3 and Table 2).

We were able to substantially improve agreement between diagnostic codes and confirmed clinical cases by removing codes with low predictive potential (particularly I38 in a secondary position), very short admissions (< 3 days) without death, and then (after excluding short admissions) readmissions within 30 days of a previous (endocarditis-coded) discharge date (for details, see Additional file 1: Extended Methods). This combination substantially improved specificity and PPV, with only a small loss in sensitivity for Duke definite/possible cases in Leeds (0.69/0.89/0.78 respectively) (Table 2). Results were broadly similar (PPV 0.77) in Oxford (Additional file 1: Table S5).

The majority of studies of endocarditis incidence use only the ICD-10 code I33.0, or I33.0 and I33.9 codes (or ICD-9 equivalents). Using I33.0 in any position had similar specificity and PPV in the Leeds data to the strategy above but with reduced sensitivity (sensitivity/specificity/PPV 0.55/0.91/0.77) (Table 2). The strategy with the highest PPV (88%) used I33.0 in the primary position alone [14, 38], but also removed short stays, readmissions and elective admissions. However, despite its high specificity (0.97), this strategy had reduced sensitivity (0.41) (Table 2 and Additional file 1: Figure S4), and hence underestimated overall incidence (Fig. 3). Including short stays, readmissions and all elective admissions with the I33.0 primary code, more similarly to studies on English HES data [14, 38], reduced the PPV to 82%.

### Incidence trends depend on specific diagnostic coding algorithms

There was strong evidence of upward trends in incidence of uncorrected endocarditis-coded admissions per 100,000 population in Leeds (annual rate ratio, aRR = 1.07 (95% CI 1.03–1.12) *p* < 0.001), whilst confirmed clinical cases occurred at much lower incidence and showed smaller incidence increases (aRR = 1.03 (95% CI 0.97–1.09) *p* = 0.31). Estimating incidence using the steps outlined above (removing codes with low predictive power, short stays and readmissions) substantially improved agreement between estimated and true incidence of endocarditis, although it similarly tended to overestimate incidence increases and suggest stronger statistical evidence to support them (Fig. 3), whether based on all codes except I38 secondary or using only the highly specific I33.0 code in the primary position (although the latter also tended to underestimate incidence). Similar estimated incidence patterns were seen in Oxford (Additional file 1: Figure S5), but as information on confirmed clinical cases was only available from 2010 to 2016 in this dataset, no comparison in trends was possible.

### Estimating incidence of streptococcal endocarditis using secondary codes can overestimate increases over time

**Fig. 4:**
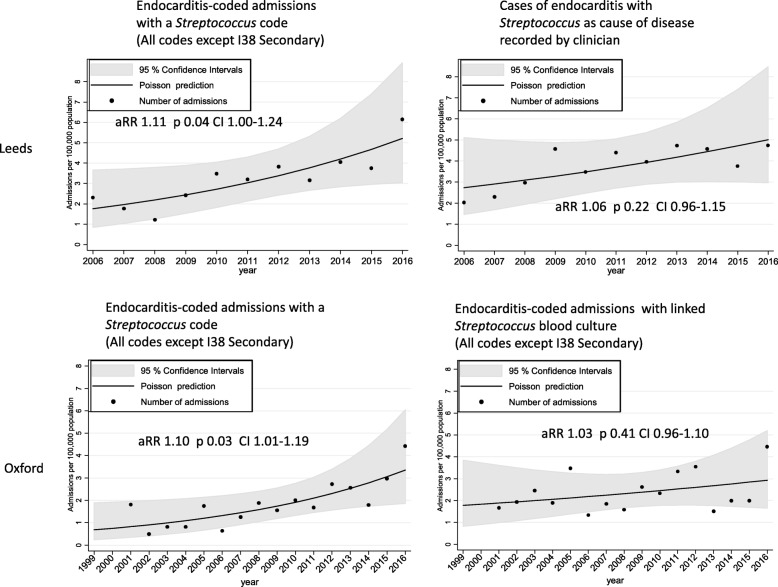
Comparison of endocarditis-coded admissions with a *Streptococcus* code, and confirmed clinical cases or blood culture data in Oxford and Leeds

**Fig. 5:**
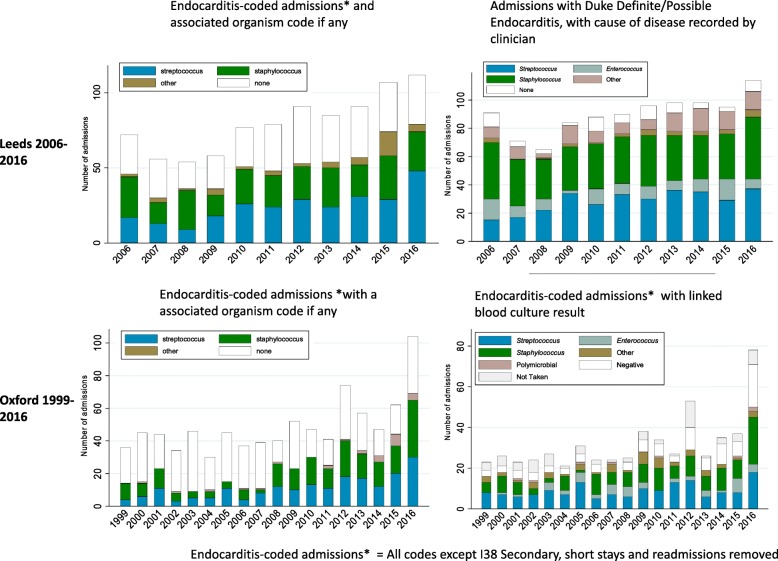
Comparison of coded organism and clinician-recorded organism (Leeds) or blood culture organism (Oxford)

Not unexpectedly, in endocarditis-coded admissions and confirmed clinical cases, the most common organisms associated with endocarditis were *Streptococcus* spp. and *Staphylococcus* spp. There are no diagnostic codes for the oral viridans group *Streptococcus* species, which are most likely to be affected by changes in dental prophylaxis, so we were unable to compare trends in these organisms. Estimating the incidence of streptococcal endocarditis based on the presence of secondary *Streptococcus* codes in endocarditis-coded admissions suggested an increase over time in both Leeds and Oxford (*p* = 0.04 and *p* = 0.03 respectively, Fig. 4). This apparent upward trend was not seen when the incidence of streptococcal endocarditis was calculated using confirmed clinical cases in Leeds (*p* = 0.22) or using information from linked blood culture results in Oxford (*p* = 0.41) (Figs. 4 and 5, Additional file 1: Figure S6 and Figure S7).

### Increased use of secondary codes over time may contribute to the apparent overestimation of streptococcal cases

In Leeds, there was moderate agreement between streptococcal codes and *Streptococcus* spp. as cause of disease. Of 314 cases judged to be of single organism streptococcal aetiology by the clinician, 201 (64%) had an associated streptococcal code (kappa = 0.56), 94 (30%) had no organism code and 19 (6%) had a different organism code (Additional file 1: Table S6; 91% (201/220) agreement where a code was given). In Oxford, overall agreement between linked blood culture results and coded organisms was similar: of 183 endocarditis-coded admissions with a linked positive streptococcal blood culture alone, 107 (58%) had a *Streptococcus* code, 68 (37%) had no code and 8 (4%) had another organism code (kappa = 0.43) (Additional file 1 Table S7; 93% (107/115) agreement where a code was given). Use of secondary/supplementary organism codes, and secondary codes overall, increased substantially during the study period in both centres (Additional file 1: Figure S8).

## Discussion

Here, we aimed to use endocarditis as a clinically relevant case study to explore the relationship between clinical cases and diagnostic codes and quantify and understand discrepancies. Investigation of the quality of coded infective endocarditis data in two large teaching hospitals, recorded between 1999 and 2016, found that different diagnostic codes vary widely in their accuracy at identifying confirmed clinical cases. Poor specificity of coding data could be explained by several legitimate coding practices; for example, the coding protocol legitimately allows diagnostic codes with the word ‘endocarditis’ to be applied to readmissions and investigations for infective endocarditis, and even to admissions with no endocarditis issues at all. We have, however, shown that the overall accuracy of coding data can be improved by careful and critical selection of codes, removal of records with implausibly short stays and removal of readmissions. The study has also shown that using secondary/supplementary codes to estimate incidence of streptococcal endocarditis can give misleading incidence trends, likely due to increasing use of such codes over time. When used, the organism codes were reasonably accurate at species level in the two centres included in this study; this suggests they could be used to assess changes in proportions of differently coded organisms over time, provided there was careful consideration of potential for large-scale changes in coding behaviour, such as incentivisation to record specific organisms, in other studies.

### Study strengths

This study, which used clinician-collected prospective data in Leeds and retrospective audit data in Oxford, based on objective clinical criteria, is the largest and most detailed study of endocarditis coding accuracy to date with 2233 patient admissions reviewed, and is the first in a UK setting. It is the first to identify and quantify the reasons for discrepancies between admissions with a diagnostic code and clinical cases. Another major study strength was the availability of detailed microbiological data on causative organism, via clinician-recorded cases in Leeds, and linked microbiological data in Oxford.

### Study limitations

Study limitations include the dual-centre nature of the study, and the limited information on confirmed clinical cases in Oxford. In Oxford, where secondary/supplementary codes were matched to blood culture data, mismatches might also be due to patients having positive blood cultures from infections other than endocarditis. The organism codes do not identify the oral viridans group streptococci, which are most relevant to changes in antibiotic policy, and we did not attempt to identify them from coding data, focussing on genus-level comparisons. This study did not set out to investigate temporal associations between changes in antibiotic prophylaxis policy and endocarditis incidence, due to limited power with only two centres, but to assess the relationship between endocarditis-coded admissions and confirmed clinical cases. Previous studies investigating temporal associations using administrative coding data have varied in their findings [12–14, 24, 27]. These studies benefit from far larger numbers than our study, although generally they have not investigated the relationship between diagnostic codes and confirmed clinical case, except for Toyoda et al. [19]. Most have used a restricted set of codes with reasonable performance in our study (Additional file 1: Table S2).

### Comparisons to other studies

Two other US [19] and Canadian [39] studies of 1673 and 119 hospitalisations, respectively, have assessed the accuracy of endocarditis diagnostic codes. Sensitivity and PPV of the ICD-9 codes equivalent to those used here (Table 1) were higher than in in our study (0.94/0.94 [19], 0.90/0.78 [39], 0.70/0.70 Leeds). Both the US [14] and Canadian [39] studies also identified the poor predictive value of ‘Endocarditis, valve unspecified’ (ICD-9 424.9, corresponding to code I38), though they did not identify the underlying cause. Previous meta-analyses of coded data to identify healthcare-associated infections have noted moderate sensitivity in detecting *Clostridium difficile* infection (pooled sensitivity 76%, specificity 99%) and surgical site infections (sensitivity 81% specificity 97%) [40]. A US study of sepsis coding compared to sepsis objective clinical criteria found that admissions with sepsis codes had increased, which was not reflected in incidence of admissions meeting sepsis clinical criteria, possibly due to changes in coding behaviour [41].

There is one other large-scale infective endocarditis study that used direct microbiological data rather than administrative diagnostic codes via three population-based surveys undertaken at different time periods [42]; it also found no increase in the proportion of cases caused by streptococci. A much smaller study of 106 admissions with infective endocarditis linked with corresponding blood cultures suggested the proportions of causative organisms were similar in coded and microbiological data [43], similar to our results.

Supplementary codes in particular may be more susceptible to changes in coding behaviour, such as incentivisation to record more secondary codes [44, 45] (so-called ‘coding depth’) or specific organisms, or availability and expertise of coding staff. However, analysis of incidence of endocarditis attributed to specific organisms differs from analysis of proportions of endocarditis with an organism code that are attributed to specific organisms. The previous study using English HES data [14] found that the proportion of endocarditis cases with any supplementary causal organism coded increased over time, particularly before 2009. Given our observations that trends in streptococcal endocarditis based on use of supplementary coding may not match that based on clinician-recorded cases, our study supports the view that using these codes is unlikely to give meaningful information on the incidence of organism-specific endocarditis. However, where changes are driven by coding depth (i.e. more codes are recorded over time, but with no specific preference for particular secondary/supplementary codes over others), proportions should be relatively unaffected.

### Implications for electronic health record study design in endocarditis

Our work suggests that studies investigating endocarditis using electronic health record data should not use the ‘I38: Endocarditis: valve unspecified’ code in the secondary position, further supporting the findings of Toyoda et al., since coding protocols allow it to be assigned to admissions featuring non-specific valve disorders entirely unrelated to endocarditis. Of note, most previous studies of endocarditis incidence did not use this code and are not affected by the issue, although at least two studies have used it [13, 46] (Additional file 1: Table S2).

Table 2 shows clearly the trade-offs between sensitivity, specificity and PPV in any coding algorithm. How these are balanced may depend on the aims of any particular study. If the goal is to maximise sensitivity to assess overall incidence levels, then inclusion of secondary codes, and potentially manual review of secondary codes with low positive predictive value, may be required, or risk missing 25–50% of cases. Where manual review is impractical, then identifying the highest sensitivity that maintains reasonable specificity and/or PPV may provide the best balance. It is important to note that whilst maximising PPV alone may appear attractive, a very strict rule can achieve high PPV whilst missing most true cases (low sensitivity), underestimating incidence and with an uncertain impact on trends. Overall, we consider that using all codes except I38 secondary provides a good balance between PPV and sensitivity (Fig. 3) in our data set.

### Clinical and policy implications

Regarding the clinical concern that infective endocarditis increased in England [14] and the USA [24] after changes in antibiotic dental prophylaxis around 2007, our work suggests that the major studies examining endocarditis incidence have not used any poorly predictive codes, but that the algorithms used could nevertheless have overestimated incidence trends by including short admissions/readmissions. In particular, moves to reduce length of stay in English hospitals have been accompanied by parallel increases in readmissions over the last decade [47] with uncertain impact.

Given the discrepant findings of electronic health record studies, work to definitively quantify the efficacy of dental prophylaxis in preventing endocarditis may require a national registry of disease to be established, as previously suggested, though these are not without their drawbacks and concerns about data quality, and require significant resources. Alternatively, despite the significant resources required, it may be that efforts to set up a large-scale individually randomised controlled trial will ultimately be required to test the benefits of antibiotic prophylaxis.

### Implications for electronic health record study design in general

Our study illustrates clearly that using diagnostic codes which appear to represent a disease entity based on their code title without any attempt to validate these codes to clinically confirmed cases can lead to very large errors if done incautiously. This has relevance beyond the field of endocarditis and is applicable to any study conducted using diagnostic codes to assess patterns of disease. Without deduplication and careful code choice, more than half the codes assigned can represent not cases, but readmissions, investigations where the presumptive diagnosis is later ruled out and past histories. A diagnostic code most definitely does not necessarily equal a clinical case. Importantly, this does not generally suggest problems with clinical coding per se, only that the current clinical coding process has different goals to epidemiology, being primarily for reimbursement and recording of hospital activity, rather than clinical diagnoses.

Secondary codes can be susceptible to changes in coding behaviour, depending on the disease entity, including measures to increase quality as well as ‘up-coding’ (choosing the code worth the most) or ‘coding inflation’ (where multiple secondary codes are used to increase reimbursement), which have been reported in the UK and other healthcare settings using these systems [44, 45, 48]. However, studies that aim to maximise inclusion of possible cases should not automatically disregard them, as a substantial proportion of confirmed clinical cases may only receive a secondary code, as in our endocarditis examples.

Recommendations for conducting observational studies using routinely collected health data already exist [49] and include detailing validation study methodology or providing references for this. In studies which use a very large selection of diagnostic codes, it may not be possible to validate every code, but at minimum, diagnostic codes that occur most commonly should be monitored over time by centre, and unexpected changes discussed with both coding and clinically trained staff. Additionally, studies using coding would benefit from a statement by authors which justifies the chosen coding strategy based on available data and highlights the limitations of their approach. Any clinical decisions made using diagnostic code-based analyses should also formally consider whether robust validation of coding has been performed and review justification for the chosen strategy.

Finally, it suggests more work is needed to explore novel methods of improving case identification using electronic health records, such as improving data linking between admissions and microbiology results [30], using natural language processing methods [50], machine learning approaches [51]or healthcare process modelling [52], and supporting efforts to share, evaluate and refine these methods [53].

## Conclusion

Our study comprehensively evaluates the accuracy of clinical coding of infective endocarditis in two UK centres. It highlights that diagnostic codes were never intended for observational epidemiology, and ‘mission creep’ in their use requires validation against other sources of data rather than the assumption that verbal descriptions are clinically meaningful. Their findings cannot be seen as definitive or replacing other research methodologies. They are useful as a relatively resource-light method of assessing issues that demand closer attention where possible, or studying issues where other research methods are infeasible. The study should serve as a learning point for anyone wishing to use diagnostic codes to assess patterns of disease, and emphasises the need for improvements in how we define clinical diagnoses using routinely collected data.

## Additional files

Additional file 1: Extended Methods. **Table S1.** Summary of studies of endocarditis incidence or features using electronic health record data or microbiological data, source of information, codes used, methods of deduplication and comparisons of codes and cases. **Table S2.** Summary of endocarditis codes used in the above studies. **Table S3.** Secondary/supplementary organism codes used and reviewed. **Figure S1.** Clinical reviews in the Leeds Endocarditis Service database, Duke status and diagnostic codes. **Figure S2.** Admissions with an endocarditis diagnosis code and selection for review: Oxford. **Figure S3.** Review of electronic prescription data in Oxford 2016 with matching to coded data. **Table S4.** Reviews and Duke status in the Leeds Service Database and matching to endocarditis-coded admissions. **Table S5.** Agreement between different combinations of endocarditis-coded admissions and confirmed clinical cases in Oxford. **Figure S4.** Sensitivity/specificity and positive predictive values for different algorithms to identify Duke definite/ possible endocarditis cases from diagnostic codes in Leeds and Oxford. **Figure S5.** Estimated endocarditis incidence in Oxford based on diagnostic coding and administrative information. **Figure S6.** Estimated endocarditis cases and causative organism from diagnostic codes compared to clinician cases, Leeds. **Figure S7.** Estimated endocarditis cases and causative organism from diagnostic codes and microbiological cultures, Oxford. **Table S6.** Coded organism vs clinician-recorded organism in Leeds Duke definite/possible cases. **Table S7.** Coded organism vs microbiology blood culture organism in all admissions with a non-I38 endocarditis code in Oxford. **Figure S8.** Coding depth and use of secondary/supplementary organism codes in Leeds and Oxford. (DOCX 2110 kb)

## Data Availability

Request for copies of the analysis code and study protocols should be addressed to nicola.fawcett@ndm.ox.ac.uk. Requests for copies of the analysis data will be considered on an individual basis. Identifiable audit data cannot be shared. Approval for data sharing for anonymised Oxford data will require approval from the Infections in Oxfordshire Research Database team. Approval for data sharing for anonymised Leeds data will require approval from the Leeds Teaching Hospital NHS Trust Information Governance team.

## Abbreviations

HES: Hospital Episode Statistics
ICD-10: International Classification of Diseases 10th Revision
Leeds: Leeds Teaching Hospital
NHS: National Health Service
NPV: Negative predictive value
Oxford: Oxford University Hospitals NHS Foundation Trust
PPV: Positive predictive value
