# Supplementary material for: ‘Caveat emptor’: the cautionary tale of endocarditis and the potential pitfalls of clinical coding data—an electronic health records study
Source: BMC Med. 2019 Sep 4;17:169. doi: 10.1186/s12916-019-1390-x (PMC6724235; doi:10.1186/s12916-019-1390-x)

**ADDITIONAL FILES**

**ADDITIONAL FILE 1: Detailed Methods**

**Additional details on classification of cases and readmissions**

In Leeds, clinical cases were prospectively identified by the Endocarditis service by clinical review of all patients with suspected infective endocarditis referred by physicians across all sites. To classify admissions as cases, readmissions or prior history, we first classified direct overlaps before admission and discharge and clinician-recorded dates. For all other admissions, a readmission was defined as an admission that did not match directly to a case, and occurred within 30 days of the discharge date of a previous case. A prior history was defined as an admission not matched to a case, that occurred more than 30 days after the discharge date of a previous case.

In Oxfordshire, a clinical case was defined at audit as an admission where a new diagnosis of Duke definite/possible endocarditis was made (or a new genuine recurrence, including recurrence in a new prosthetic valve after surgery for infective endocarditis). A readmission was classified if the admission was for management of complications of a known prior episode of endocarditis, and the readmission date was within 30 days of a previous discharge date for endocarditis. A patient was defined as having a new, true second case of endocarditis (as opposed to failure of treatment/complication) if treatment for the prior episode had finished, and the managing clinician documented they felt it was a new case in the same patient. In the few cases where the patient was seen briefly as possible endocarditis and then sent home to await blood cultures, then readmitted later when cultures were flagged positive, the review counted the second admission as a clinical case, and the first, short admission as a readmission (technically a preadmission).

1. **Oxford audit and review of endocarditis coding description**

In Oxford, electronic and paper notes from endocarditis-coded admissions 2010-2016 were retrospectively reviewed in an audit of endocarditis coding accuracy. Admissions were assessed as to whether the features a case of endocarditis were present according to modified Duke Criteria^1^ by a clinician (BY, LP, NJF) (Additional File 6: Figure S2). It was also recorded whether this was a readmission, complication, or featured a past history of infective endocarditis, if infective endocarditis was investigated and excluded, or no infective endocarditis was evident in medical records. Ambiguous cases were reviewed by a second and if necessary third clinician, to achieve consensus by majority. A random set of 41 admissions were reviewed by two clinicians. There were two discrepancies where one reviewer had misrecorded a staphylococcal wound culture as being a blood culture, and another where one reviewer had not seen a third blood culture. Findings were reviewed with the clinical coding team. Five admissions with an endocarditis code and no history of endocarditis were reviewed in detail, with the full coding process followed including use of both international and England-based^2,3^ coding guidance and alphabetical indices^4^.

1. **Normalisation to Population data**

Population data was obtained from the Office of National Statistics, available for 2001-2016. Population data for the Leeds Local Authority District was used to represent catchment area for Leeds Teaching Hospital NHS trust, with 2016 data on population served (781,087) matching closely to quoted figures from official Trust reports of 780,000 in 2016^5^. The trust takes a small number of referrals from other hospitals in the region, but this information was not recorded in the Leeds Endocarditis Service Database. Using Leeds population data only may thus slightly over-estimate incidence per population head, but given population growth was similar in Leeds and surrounding areas^6^, should not affect incidence trends.

For Oxfordshire data, population was evaluated as population in Oxford, South Oxfordshire, West Oxfordshire, Vale of White Horse and Cherwell Local Authority Districts (collectively part of Oxfordshire county), all of which have hospitals where Oxford University Hospitals is the tertiary referral service, and combined population data (678, 484) of closely matches trust reports for 2016/17 (655,000)^7^.

Where incidence of endocarditis-coded admissions with blood culture data analysis was performed, results of blood cultures taken at time of admission were only available for patients admitted directly to the John Radcliffe Hospital or Horton Hospital. In this case, number of admissions was normalised to population of Oxford alone.

1. **Method for assigning I38 code**

The findings regarding the use of I38 for cases without any mention of endocarditis in clinical notes was discussed with the Clinical Classifications Service, UK, the definitive source of clinical coding guidance who set the national standards for ICD-10 used by the NHS, who responded:

*“The ICD-10 4th Edition is a classification, by the very nature of a classification the ICD-10 4th Edition code I38.X Endocarditis, valve unspecified, can be assigned when there is no mention of endocarditis in the medical record, but the ICD-10 alphabetical index directs you to assign a code from this category. Therefore, a coder would be correct to assign code I38 when indexing a documented diagnosis which leads the coder to assign code I38, even when the term endocarditis is not documented within the medical record.​"*

*-Clinical Classifications Service, Health and Social Care Information Centre, UK*

1. **Improving agreement between admissions with an endocarditis diagnostic code and Duke definite/possible endocarditis cases**

Improved agreement between endocarditis-coded admissions and Duke definite/possible cases could be achieved by iteratively addressing the legitimate types of discrepancies identified in the main analysis.

First, given the low positive predictive value, we excluded I38 where this occurred as a secondary code only. Second, we excluded any patients with a length of stay of 2 days or fewer (discharge date minus admission date), that were discharged alive (see main Methods). Third, we removed coded admissions where the patient had a previous admission with an endocarditis code with admission date within 30 days of the previous discharge date. By performing the removal of admissions in this order meant that patients who were admitted twice, with one short and one long stay, would still be included if the short stay preceded the long stay. Fourth, we also considered removing elective admissions (admission method starting with “1”). We noted that a number of Duke Criteria definite or possible cases had elective status. On review, these were predominantly patients admitted for elective procedures who then developed endocarditis as an inpatient. Primary analyses therefore did not exclude these patients.

1. **Designing the fuzzy text search for endocarditis**

For all electronic antibiotic prescriptions, Oxford implemented a mandatory ‘Indication’ field to record reason for administration in accordance with UK Department of Health Guidance^8^

We searched all prescribing records January-December 2016 for the term “endocarditis” and compared it to confirmed clinical cases identified by the audit of admissions with an endocarditis code. We converted all strings into lowercase. We identified common misspellings like “rndocarditis” or “endocaritidis” that our initial text search would have missed. Our best match was using a search of the string “ndoca” which incidentally resulted in no ‘false’ identifications.

We subsequently reviewed all patient records with an indication containing the text string “ndoca” “ie”, “prosthetic valve”, “aortic root” and “vegetation”. “ie” identified a number of infective exacerbations e.g. ie asthma, iecopd, so we subsequently restricted the text string to “ie” without “copd” “ild” “asthma” or “cf”.

There were 9 ‘true IE’ cases that were missed through our text search, but it was not possible to design a text search for these, as they had indications “mssa bacteraemia”,” liver abscess”, “lung abscess”, “cerebral abscess”, and “infective endo. 18/8 dose”

References:

1. Li JS, Sexton DJ, Mick N, et al. Proposed modifications to the Duke criteria for the diagnosis of infective endocarditis. *Clin Infect Dis.* 2000;30(4):633-638.

2. *National Clinical Coding Standards ICD-10 4th Edition.* NHS Classifications Service, Health and Social Care Information Centre, UK; 2013.

3. ICD-10-CM Official Guidelines for Coding and Reporting. In: U.S. Department of Health and Human Services; 2017.

4. ICD-10 Alphabetical Index Fourth edition. In: World Health Organisation; 2013.

5. Trust OUHNF. *Annual Report and Accounts for 1 Oct 2015 - 31 March 2016.*

6. Population Estimates for UK, England and Wales, Scotland and Northern Ireland. In: The Office for National Statistics U, ed2001-2016.

7. Oxford University Hospitals NHS Trust Quality Report. In: Care Quality Commission; 2014.

8. Commissioning for Quality and Innovation (CQUIN) Guidance for 2017-2019. In: NHS England; 2017.

**Table S1** Summary of studies of endocarditis incidence or features using electronic health record data or microbiological data, source of information, codes used, methods of deduplication and comparisons of codes and cases

| Author and Year | Study population | Finding | Identification of cases | Deduplication /other | Comparison of codes to true incidence |
| --- | --- | --- | --- | --- | --- |
| Assessment of change associated with guidelines | | | | | |
| Tubiana ^9^2017 | Adults in France with codes indicating prosthetic valve 2008-2014 | No increased rate of oral streptococcal endocarditis in individuals exposed to invasive dental procedure with or without prophylaxis compared to non-exposed. | Primary | First hospitalization with infective endocarditis as defined only | Refers to Toyoda et al for choice of codes used |
|  | Source: French health insurance and hospital discharge database | Exposure to invasive procedure more frequent in case vs control periods 5.1% vs 3.2%. | I330 I339 |  |  |
|  | 138 876 individuals, 52281 had invasive dental procedure. 267 infective endocarditis with oral streptococci. |  | Streptococcus: |  |  |
|  |  |  | A40.8, A40.9, A49.1, B95.4, B95.5 |  |  |
|  |  |  | Presence of valve prosthesis |  |  |
|  |  |  | Z95.2, T82.6 T82.0 |  |  |
|  |  |  |  |  |  |
| Bates^10^ 2017 | Children <18 years hospitalized with infective endocarditis at 29 centres in US 2003-2014 | Interrupted Time Series analysis: increasing trend pre-guideline change, no difference in rate of change post guidelines. | Primary or Secondary | First(index) hospitalization with infective endocarditis as defined only | No |
|  | 841 cases identified from paediatric health information system database. | Similar analysis in children with congenital heart disease found similar results. | 4210 |  |  |
|  |  |  | Plus |  |  |
|  |  |  | antistreptococcal antibiotics >7 days (vancomycin/penicillin/ceftriaxone monotherapy or vanc/gent combi or cef/pen and gent- American Heart Association guidelines) |  |  |
|  |  |  | Also considered congenital heart disease, codes listed in reference Jenkins et al^11^ |  |  |
| Bikdeli^12^ 2013 | Older adults: >=65 yrs with principal/secondary diagnosis of endocarditis in National NIS database 1999-2009. 262,658 individuals hospitalized with endocarditis. | Adjusted hospitalisation rate increased 1999-2005, then decreased, with the decline continuing after 2007. No change in adjusted mortality, and trends consistent across subgroups. | Primary or secondary | If multiple hospitalisations in given year (18%) random hospitalization used (patient unit of analysis) | No |
|  |  |  | 4210 4211 4219 **4249** |  | In discussion-reference |
|  |  |  | Excluded if 996.91code used in any position (intracardiac device) . |  | Fedeli^13^, Cabell^14^, Mendiratta^15^, Rogers^16^ Day^17^ |
|  |  |  |  |  |  |
| DeSimone^18^ 2012 | All hospital discharges with streptococcal infective endocarditis in National NIS database 1999-2009. Approx... 135,000 hospitalisations | Incidence calculated in 3x 3 year time intervals | 421 | All hospital discharges | No |
|  |  |  |  | No deduplication |  |
|  |  | No change in viridans group strep incidence in 3 year period post guideline nationally or in smaller subgroup. | Streptococcus codes: |  |  |
|  | +Prospective study using Mayo Clinic Endocarditis registry, Viridans-group modified Duke criteria Endocarditis 1999-2010, total 150 cases, of which 22 cases were viridans group strep | Overall decline in incidence noted- authors speculate due to declining rheumatic heart disease | streptococci unspecified: 041.00; and other streptococci: 041.09. |  |  |
|  |  |  | Excluded: |  |  |
|  |  | Rate of incidence National 1999-2002: 3.19/100,000 | strepgroup A: 041.01; |  |  |
|  |  | Local 1.7-3.5 cases/100.000. | strep group B: 041.02; strep group C: 041.03; enterococcus group D: 041.04; streptococcus group G: 041.05. |  |  |
| Duval^19^ 2012 | 3 population-based surveys 1991, 1998 2008, 993 cases. Verified by expert team and microbiology lab results | No increase in overall infective endocarditis incidence or in oral Streptococcal infective endocarditis. Increase in Staphylococcal infective endocarditis in patients with native valve disease | N/A | All cases as recorded by healthcare professionals | No |
| Van den Brink^20^ 2017 | Netherlands national insurance healthcare database 2005-2011 5213 patients hospitalized with endocarditis | Increase in cases from 30/100,000 to 63/100,000. Before/after European Society of Cardiology Guideline change streptococci 31-53% | Netherlands-specific endocarditis code for insurance | Not specified –appears first national hospitalization per patient counted. (reference to avoiding counting twice for transfers) | No |
|  |  |  |  |  |  |
|  |  |  |  |  |  |
|  |  |  |  |  |  |
| Dayer^21^ 2015 | 2000-2013 UK National hospital episode statistics (HES) database all UK patients. | Increase in infective endocarditis cases/month from 2008 above projected historical trend in both higher and lower risk individuals. | I33 Primary | Use of superspells (deduplicated for transfers) | “Although these data are subject to error, they have been shown, for example, to provide more reliable and complete data capture for vascular surgery than a UK national research database specifically designed for that purpose” |
|  |  |  |  |  |  |
|  |  |  |  | Exclusion of waiting list cases (based on preliminary analysis) |  |
| Keller^22^ 2017 | Federal Statistical office of Germany | Increase in prevalence per 100,000: 2006-2010 9.5-10.6, then larger increase 2011-2014 11.1-14.4. | ICD-10: I33 | No mention of deduplication. | No |
|  | 94,364 patients | Increasing prevalence of streptococcus and staphylococcus endocarditis, percentage streptococcus 20.8% staphylococcus 21.9% | Streptococcus: I33 + B95.0-B95.5 |  |  |
|  | ‘Annual number of inpatients diagnosed with infective endocarditis’ | Increasing number of valvular surgeries | Staphylococcus I33+B95.6-B95.8 |  |  |
|  |  |  |  |  |  |
| Pant^23^ 2015 | Healthcare cost and utilization project Nationwide Inpatient Sample(NIS) Database USA 2000-2011. | Increase in incidence of hospitalisations 2005-11, from 11/100,000 to 15/100,000. Increase in all causes (Staphyococcal, Streptococcal, Gram Negative, fungal, absolute and percentage – likely due to decreasing number of no-secondary-organism code cases). | 4210 (acute and subacute bacterial endocarditis), 4211 (acute and subacute infective endocarditis in diseases classified elsewhere – e.g. Q fever), 4219 (acute endocarditis, unspecified) | All hospitalisations | Note limitations of multiple admissions per patient (NIS data did not allow deduplication by patient) |
|  | 457,052 hospitalisations | Both staphylococcal and streptococcal cases increasing, but rate of increase of streptococcal more post-guidelines (disputed in correspondence) | Staphylococcus aureus endocarditis: 03811, 03812, 04111, 04112, 0381, 03810, 03819, 0411, 04110, 04119 |  | Acknowledge validation study limitation. Quote Fedeli et al pilot study with PPV 81% |
|  |  |  | Streptococcal endocarditis: 0380, 0382, 0410, 04100, 04101, 04102, 04103, 04104, 04105, 04109 |  |  |
|  |  | Piecewise/segmented regression analysis performed. | Gram negative endocarditis: 04185, 0384, 03840, 03841, 03842, 03843, 03844, 03849, 0413, 0414, 0415, 0416, 0417 |  |  |
|  |  |  | Fungal endocarditis: 1160, 1154, 11281 |  |  |
| Bor^24^ 2013 | Agency for healthcare research and quality NIS 1998-2009. | Organisms coded for 62.3% of infective endocarditis discharges in 1998, 69.4% in 2009. Note likelihood of undercoding. Polymicrobial 4.2% | 4210 4211 4219 03642 09884 11281 1154. | Number of hospital stays. | No |
|  |  |  | “Did not include 0.2% of cases where endocarditis was not in the first 15 diagnoses” | Deduplication for transfers: |  |
|  |  | Most common organism S. aureus, increase in proportion from 24.0-32% of infective endocarditis cases. No clear increase trend in Streptococci. | For organisms, see appendix* | Numbers of transferred patients calculated and subtracted from numbers. |  |
|  |  |  |  | Acknowledge they will include readmissions. |  |
| Mackie^25^ 2016 | Canadian Institute for Health Information 2002-2013, | Piecewise linear regression model with 2 regression lines, 2 trends before and after guidelines with change point analysis No change in slope at 2007. Increase in trend seen 2011 with decrease in trend of streptococci | Primary ICD-9 421.0-421.9 or ICD-10 I33.0-I33.9) | All hospitalisations. | No, but discussion references Fedeli and Schneeweiss |
|  | 8055 hospitalisations with a code of infective endocarditis. | Suggest aging population and more devices (more pacemakers and defibrillators) | Organism: |  | and good quality of Canadian data |
|  |  | Noted 73.8% had coexisting diagnosis. | ICD-9CM 041.x, ICD-10 B95.x) and sepsis codes (eg, ICD-9CM 038.x, ICD-10 A41.x) | Hospitalisations within 24hrs of patient being discharged from another acute care hospital- transfer and analysed as single hospitalisation |  |
|  |  | Rates 0.05 hospitalisations per month per 10 million |  |  |  |
| Toyoda ^26^: assessment of incidence 2017 | 75,829 patients with first episode of endocarditis in US state databases 1998-2013 (California and new York state). “patients with de novo endocarditis” | Standardized annual incidence stable: 7.8 /100,000. Native vale disease decreased, prosthetic valve disease increased, device related disease increased. Healthcare associated nosocomial decreased proportionally. No increase in oral streptococcal endocarditis. Crude mortality stable, adjusted mortality increased. | Primary or secondary | First admission only used. Repeat admissions and readmissions excluded. | Extensive validation in additional |
|  |  |  | 4210 4211 4219 11281 03642 09889 11504 11514 11594, |  | Note other studies with different findings may be affected by recurrences, relapses and transferred cases, and less specific ICD9 codes. |
|  |  |  | Oral streptococcus |  |  |
|  |  |  | 04100 04109 |  |  |
|  |  |  | Streptococcus |  |  |
|  |  |  | 0380 0382 0410 04100 04101 04102 04103 04104 04105 04109 0412 |  |  |
|  |  |  | Staphyloccoccus aureus |  |  |
|  |  |  | 03811 03812 04111 04112 |  |  |
|  |  |  | Staphylococcus |  |  |
|  |  |  | 0381 03810 03811 03812 03819 0411 04111 04112 04110 04119 |  |  |
|  |  |  | MRSA |  |  |
|  |  |  | 03812 04112 (after 2008) 03811 04111+v090 or V091 (pre 2008) |  |  |
| Toyoda^26^ additional: coding accuracy 2017 | Mount Sinai data warehouse discharge data, text search ‘endocarditis’ or ‘vegetation’, or ICD9 codes listed 2013-2014. 1673 hospitalisations, 515 hospitalisations, 283 patients with infective endocarditis. | Mount Sinai: | Primary or secondary |  |  |
|  |  | Sens/Spec/PPV | 4210 4211 4219 11281 03642 09889 11504 11514 11594, |  |  |
|  | Further code validation at 5 other facilities | Primary | Included, low specificity noted |  |  |
|  |  | 21.1/99.9/96.6 | 42490 42491 42499 |  |  |
|  |  | Primary and Sec |  |  |  |
|  |  | **93.6/99.9/93.9** |  |  |  |
|  |  | Prim minus 4249x |  |  |  |
|  |  | 95.5/99.9/80.1 |  |  |  |
|  |  | Prim minus 4249x 2003-2005 PPV 89 (131 pts ) |  |  |  |
|  |  |  |  |  |  |
|  |  | Also reviewed types of disease and organisms in 155 pts 2013/14. |  |  |  |
|  |  |  |  |  |  |
|  |  | 5 separate hospitals, PPV 90, 93, 90, 90, 95, 97. (total 166) |  |  |  |
| Sakai-Bizmark^27^ | HCUP Heathcare Cost and Utilization Project NIS AHRQ. Patients<18 yrs, | Incidence 4.2 per 100,000. | 4210 4211 4219 | No deduplication due to use of NIS data, noted in discussion. | “Our initial review of data revealed that using only the principal diagnosis code for infective endocarditis may significantly decrease selection of true cases, at times finding pathogen as the principle diagnosis, and infective endocarditis as the secondary. “ |
|  |  | Post guideline change increased likelihood of streptococcal (23.8% vs 32.0%,) and Virdans group streptococcal (VGS) cases (17.6% vs 24.2%) 70% cases had pathogen code, | Primary position, or |  |  |
| 2017 update | 3748 discharges | 65% preguideline, 75% post guideline. | secondary or tertiary position if primary/secondary code a relevant infective code |  | Also covered in discussion |
|  | 82% primary, 15% secondary 3 % tertiary. | Staph aureus most common (34%), streptococcus (27%) Overall increase in incidence, no change in trend pre and post guidelines. |  |  |  |
|  |  | Increasing trend in VGS in older patients subgroup | following: (1) Pathogen: described below; (2) Sepsis: 995.9; (3) Bacteremia: 790.7; (4) Septic Shock: 785.52; (5) Device Complication: 996–999; and (6) Pneumonia: |  |  |
|  |  |  |  |  |  |
|  |  | Note Pasquali et al |  |  |  |
|  |  | Indexed to hospital admissions, not population numbers |  |  |  |
| Cresti^28^ 2017 update | 1807 Referrals to tertiary hospital, Italy 1998-2014 for suspected infective endocarditis entered prospectively into database. | Incidence 4.6/100,000 population head, increasing linearly. | Not specified ‘endocarditis code’ ICD-9 | Covered in discussion. | Data provided: Total 173 patients (ie database identified 6 extra, sensitivity 97%, coding identified 143/173 84%) |
|  | 167 definite infective endocarditis (Duke criteria). | Staphylococcus aureus 25%, Coagulase negative Staphylococci (CNS) 22%. |  |  | 194 admissions, of which 25(13% had no criteria for definite infective endocarditis, 28 (14%) were readmissions. |
|  | Additionally, healthcare system database interrogated for discharge records with ICD9 codes – 194 patients, definite infective endocarditis in 143. | Staphylococcal disease associated with intravenous drug use (IVDU) and septic shock. Culture negative 11%. |  |  | Discuss limitations of discharge-coding only studies |
|  | Total 173 patients |  |  |  |  |
| Gupta^29^ | NIS database | Incidence 0.43/100,000. | Primary and secondary diagnoses, ICD-9 | None. | Note limitations of ICD-9 coding use, no duke criteria, and readmissions/transfers. |
| 2017 update | 3840 hospitalisations of children <20yrs. | 30% of cases culture negative. | 421.X | Covered in discussion |  |
|  |  | Staphylococcal 43% Streptococcal 40%. Among culture-positive patients, increased proportion of Streptococcal cases ie, decrease in staphylococcal ie. | 09884, 03642, 11281, 11504 11514 11594 *** |  |  |
|  |  |  |  |  |  |
|  |  |  |  |  |  |
| Pasquali^30^ 2012 | 1157 infective endocarditis cases in Children <18yrs 2003-2010 at 37 centres in Paediatric Health Information Systems Database USA | Raw number of cases did not change over time. Indexing to number of hospital admissions showed decline in admissions. No population comparison. | 4210 in any position plus 7 days of antibiotics within 7 days of admission. | First (index) hospitalization included | No |
|  |  |  | oral streptococcal species (ICD-9 codes 038.0, 038.3, 041.00, 041.03, 041.05, 041.09) |  |  |
|  |  |  | Those with codes for Group A, B, or D streptococcus, pneumococcus, or staphylococcus species were excluded from this classification |  |  |
| Thornhill^31^ 2018 | Marketscan US healthcare insurance database (Medicare and Medicaid) 240 million individuals Jan2000-Aug2015 | 20,340 episodes of IE. Poisson regression modelling before, during and after guideline change suggested decreasing rate prior to guideline change, with subsequent continuing downward trend with reduced rate of decrease. IE cases/month/100,000 expected from pre-guideline trends 11.04, actual observed 30.57 cases/month/100,000 | ICD-9 code 421.0, 421.1, or 421.9, primary or secondary discharge diagnoses | Continuous episodes counted once as per Thornhill 2011^32^. New episodes distinguished from readmissions if >6 months apart as new episodes. | Not done |
|  |  |  |  |  |  |
|  |  |  |  |  |  |
| Assessments of coding accuracy | | | | | |
| Fedeli ^13^2011 | 1863 subjects hospitalized for infective endocarditis in Italy Veneto region | Increasing trend in infective endocarditis 2000-2008. Rate 4.4/100,000 | 421.x 98.84(gonococcal) 112.81 candidal | Day case excluded. Pre-2000 excluded. | Note lack of validation of ICD9 codes. |
|  | Linked mortality, linked microbiological database. 502 had organism code, 106 had blood culture data. |  | Used 38.c or 41.x microbiological codes but rarely reported. | First hospitalization 2000-2008 counted. | Pilot investigation described in discussion: at a single Veneto hospital chart review of discharges and extraction of codes (including 421.x 98.84, 112,81, 93.2 391.1 and **424.9x** **996.61**(T826 and T827) (note typo in the manuscript) against Duke criteria infective endocarditis. PPV 91/123 81% PPV, sensitivity 91/98 93%. |
| Schneeweiss^33^ 2007 | Overall accuracy of bacterial infection coding, 127 patients with coded infections selected for review | Overall specific PPV 80%/infection generally 90% | 421.x used | NA | NA |
|  |  | Endocarditis: 14/19 were true, with PPV 74% | Considered and excluded 036.42, 093.2x 098.84, 391.1, 397.9, 421.0 421.9 422.92 |  |  |
|  |  |  |  |  |  |
| Sunder^34^ 2015 | French Regional Hospital Discharge Database (part of French Hospital Discharge Database) 2007-2009 2.53 million inhabitants. | First: | Any position: | Ambulatory stays <24hrs excluded |  |
|  | First: | PPV 87.4% 128/173 definite, 44 possible 1 insufficient data. | I33 I38 I39 T826 | One episode per patient (justified as 2 year study, assumed same infective endocarditis) |  |
|  | 243 stays 198 patients randomly selected and reviewed by ID specialist. | Second: | Extensive codes see appendix** |  |  |
|  | 173 patients had infective endocarditis duke criteria. | NPV 99.4% PPV 100% sens 90% spec 100% | Codes for infection due to pathogens responsible for infective endocarditis: A40 A48 A78 A395 A410 A411 A412 A413 A414 A415 A418 A419 A448 A449 A478 A479 A490 A491 A493 A498 A499 B376 B377 B960 B961 B962 B963 B964 B965 B966 B968 |  |  |
|  | Second: | These values used to adjust incidence estimates: |  |  |  |
|  | 492 random patients post valve surgery, TOE or PPM removal | infective endocarditis incidence 45.8 case per million |  |  |  |
|  |  | Microbiological code 309 patients (53.5%) 11,2% nonspecific bacterial infection, Streptococci 49% staphylococci 36%. |  |  |  |
| Note: PPV=positive predictive value. NA Not applicable | | |  |  |  |

**Table S2** Summary of Endocarditis codes used in the above studies

| Author | Other | I33.0 Primary | I33.0 Secondary | Other Primary | Other Secondary | I38 Primary | I38 Secondary | Organism codes |
| --- | --- | --- | --- | --- | --- | --- | --- | --- |
| Tubiana ^8^2017 |  | Yes |  | I339 |  |  |  | yes |
|  |  |  |  |  |  |  |  |  |
| Bates^9^ 2017 |  | yes | yes |  |  |  |  |  |
|  |  |  |  |  |  |  |  |  |
| Bikdeli^11^ 2013 |  | yes | yes | 4211 | 4211 | 4249 | 4249 |  |
|  |  |  |  | 4219 | 4219 |  |  |  |
| DeSimone^17^ 2012 |  | yes | yes |  |  |  |  | yes |
|  |  |  |  |  |  |  |  |  |
| Duval ^18^ 2012 | survey |  |  |  |  |  |  |  |
| Van den Brink^19^ 2017 | Netherlands specific codes |  |  |  |  |  |  |  |
| Dayer^20^ 2015 |  | yes |  |  |  |  |  |  |
|  |  |  |  |  |  |  |  |  |
| Keller^21^ 2017 |  | yes | yes |  |  |  |  | yes |
|  |  |  |  |  |  |  |  |  |
| Pant^22^ 2015 |  | yes | yes | 4211 | 4211 |  |  | yes |
|  |  |  |  | 4219 | 4219 |  |  |  |
| Bor^23^ 2013 |  | 4210 | 4210 | 4211 4219 03642 09884 11281 1154. | 4211 4219 03642 09884 11281 1154. |  |  | yes |
| Mackie^24^ 2106 |  | I33 or 4210 |  | 4219/I339 |  |  |  | yes |
| Toyoda ^25^: assessment of incidence 2017 |  | yes | yes | yes | yes | yes |  | yes |
| Toyoda^25^ additional: coding accuracy 2017 |  | yes | yes | yes | yes | yes | yes |  |
|  |  |  |  |  |  |  |  |  |
| Sakai-Bizmark^26^ 2017 |  | yes |  | Yes 4211 4219 |  |  |  |  |
|  |  |  |  | If prim/sec code infective code |  |  |  |  |
| Gupta et al^28^ |  | yes | yes | 09884,03642, 11281, 11504 11514 11594 | 09884, 03642, 11281, 11504 11514 11594 |  |  | yes |
| 2017 update |  |  |  |  |  |  |  |  |
| Pasquali ^29^ 2012 |  | Yes | Yes |  |  |  |  | yes |
|  |  | plus abx | Plus abx |  |  |  |  |  |
| Fedeli ^12^2011 |  | yes | yes | yes | Yes |  |  |  |
|  |  |  |  |  |  |  |  |  |
| Schneeweiss^32^ 2007 |  | yes |  |  |  |  |  |  |
|  |  |  |  |  |  |  |  |  |
| Sunder^33^ 2015 |  | yes | yes | I39 T826 | I39 T826 | yes | yes | yes |
|  |  |  |  |  |  |  |  |  |
| Thornhill^30^2018 |  | yes | yes | 4211 4219 | 4211 4219 |  |  |  |
|  |  |  |  |  |  |  |  |  |
| Note: abx=antibiotics | |  |  |  |  |  |  |  |

**Table S3** Secondary/supplementary organism codes used and reviewed

| icd10 code | icd10 description |
| --- | --- |
| mrsa | |
| U80.1 + other staph code | Methicillin resistant agent |
| Staphylococcus unspecified | |
| A49.0 | staphylococcal infection, unspecified site |
| A41.2 | SEPSIS DUE TO UNSPECIFIED STAPHYLOCOCCUS |
| A41.2 | SEPSIS DUE TO UNSPECIFIED STAPHYLOCOCCUS |
| A41.1 | SEPSIS DUE TO OTHER SPECIFIED STAPHYLOCOCCUS |
| B95.8 | UNSPECIFIED STAPHYLOCOCCUS AS THE CAUSE OF DISEASES CLASSIFIED ELSEWHERE |
| B95.8 | UNSPECIFIED STAPHYLOCOCCUS AS THE CAUSE OF DISEASES CLASSIFIED ELSEWHERE |
| B95.7 | OTHER STAPHYLOCOCCUS AS THE CAUSE OF DISEASES CLASSIFIED ELSEWHERE |
| Staphylococcus aureus | |
| A41.0 | sepsis due to Staphylococcus aureus |
| Streptococcus |  |
| B95.5 | UNSPECIFIED STREPTOCOCCUS AS THE CAUSE OF DISEASES CLASSIFIED ELSEWHERE |
| B95.4 | OTHER STREPTOCOCCUS AS THE CAUSE OF DISEASES CLASSIFIED ELSEWHERE |
| A49.1 | Streptococcal infection, unspecified site |
| A40.8 | Other streptococcal sepsis |
| A40.9 | STREPTOCOCCAL SEPSIS, UNSPECIFIED |
| A40.3 | SEPSIS DUE TO STREPTOCOCCUS PNEUMONIAE |
| B95.5 | UNSPECIFIED STREPTOCOCCUS AS THE CAUSE OF DISEASES CLASSIFIED ELSEWHERE |
| B95.0 | STREPTOCOCCUS, GROUP A, AS THE CAUSE OF DISEASES CLASSIFIED ELSEWHERE |
| B95.1 | STREPTOCOCCUS, GROUP B, AS THE CAUSE OF DISEASES CLASSIFIED ELSEWHERE |
| B95.4 | OTHER STREPTOCOCCUS AS THE CAUSE OF DISEASES CLASSIFIED ELSEWHERE |
| B95.2** | STREPTOCOCCUS, GROUP d, AS THE CAUSE OF DISEASES CLASSIFIED ELSEWHERE |
| B95.3 | STREPTOCOCCUS PNEUMONIAE AS THE CAUSE OF DISEASES CLASSIFIED ELSEWHERE |
| A40.0 | Sepsis due to Streptococcus, group A |
| A40.1 | Sepsis due to Streptococcus, group B |
| A40.2 | Sepsis due to Streptococcus, group D |
| A40.3 | Sepsis due to Streptococcus, group D |
| A40.8 | Other streptococcal sepsis |
| A40.9 | streptococcal sepsis, unspecified |
| B95.X | Streptococcus, Staphylococcus, and Enterococcus as the cause of diseases classified elsewhere |
| Codes that feature only US specialised coding guidance (ICD-10-CM 2010)^34^, not in UK datasets | |
| B9562 | METHICILLIN RESISTANT STAPHYLOCOCCUS AUREUS INFECTION AS THE CAUSE OF DISEASES CLASSIFIED ELSEWHERE |
| A4102 | SEPSIS DUE TO METHICILLIN RESISTANT STAPHYLOCOCCUS AUREUS |
| Z1611 | RESISTANCE TO PENICILLINS |
| Z1610 | RESISTANCE TO UNSPECIFIED BETA LACTAM ANTIBIOTICS |
| Z1612 | EXTENDED SPECTRUM BETA LACTAMASE (ESBL) RESISTANCE |
| Z1619 | RESISTANCE TO OTHER SPECIFIED BETA LACTAM ANTIBIOTICS |
| A4101 | SEPSIS DUE TO METHICILLIN SUSCEPTIBLE STAPHYLOCOCCUS AUREUS |
| B9561 | METHICILLIN SUSCEPTIBLE STAPHYLOCOCCUS AUREUS INFECTION AS THE CAUSE OF DISEASES CLASSIFIED ELSEWHERE |
| B9562 | METHICILLIN RESISTANT STAPHYLOCOCCUS AUREUS INFECTION AS THE CAUSE OF DISEASES CLASSIFIED ELSEWHERE |
| A4101 | SEPSIS DUE TO METHICILLIN SUSCEPTIBLE STAPHYLOCOCCUS AUREUS |
| B9561 | METHICILLIN SUSCEPTIBLE STAPHYLOCOCCUS AUREUS INFECTION AS THE CAUSE OF DISEASES CLASSIFIED ELSEWHERE |
|  | **Note: From 1^st^ April 2016, B952 became: STREPTOCOCCUS, GROUP d, AND ENTEROCOCCUS AS THE CAUSE OF DISEASES CLASSIFIED ELSEWHERE. The ICD-9 system has had Enterococcus as a separate code 041.04, which maps to B95.2. The OUH coding team had received guidance to code Enterococcus as B95.4 –*a Streptococcus* code. |

**Figure S1** Clinical reviews in the Leeds Endocarditis Service database, Duke status and diagnostic codes


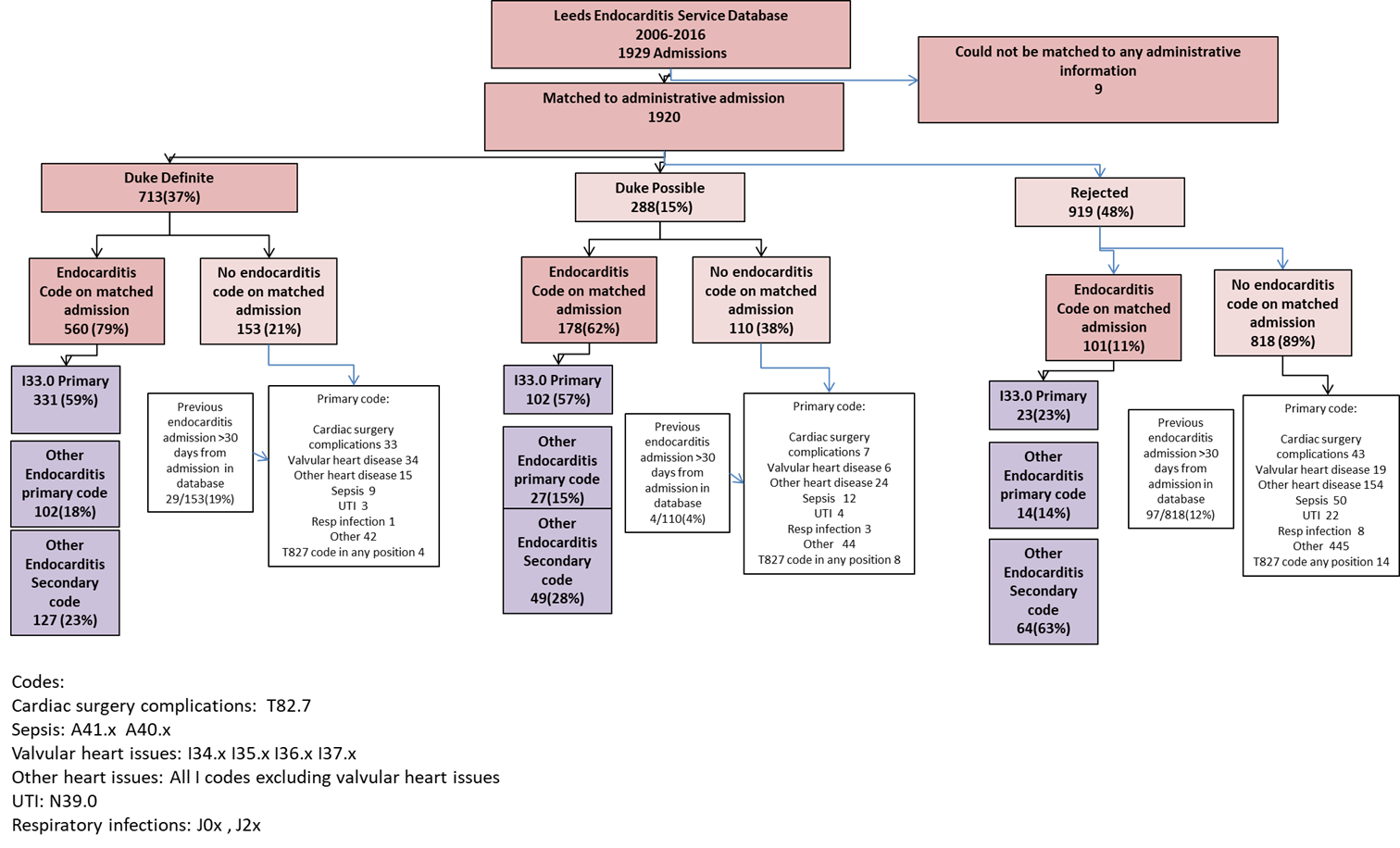


**Figure S2** Admissions with an endocarditis diagnosis code and selection for review: Oxford


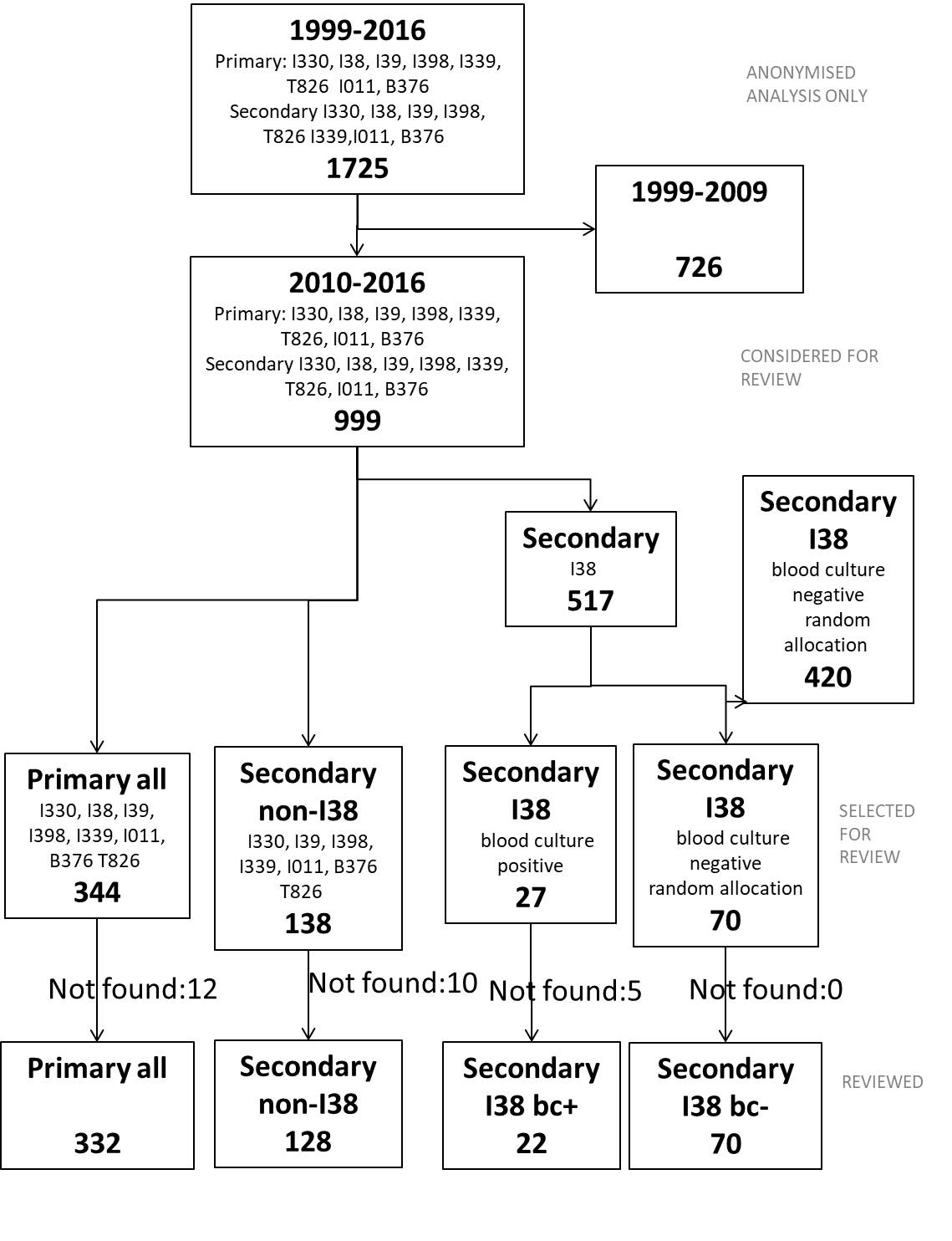


**Figure S3** Review of electronic prescription data in Oxford 2016 with matching to coded data


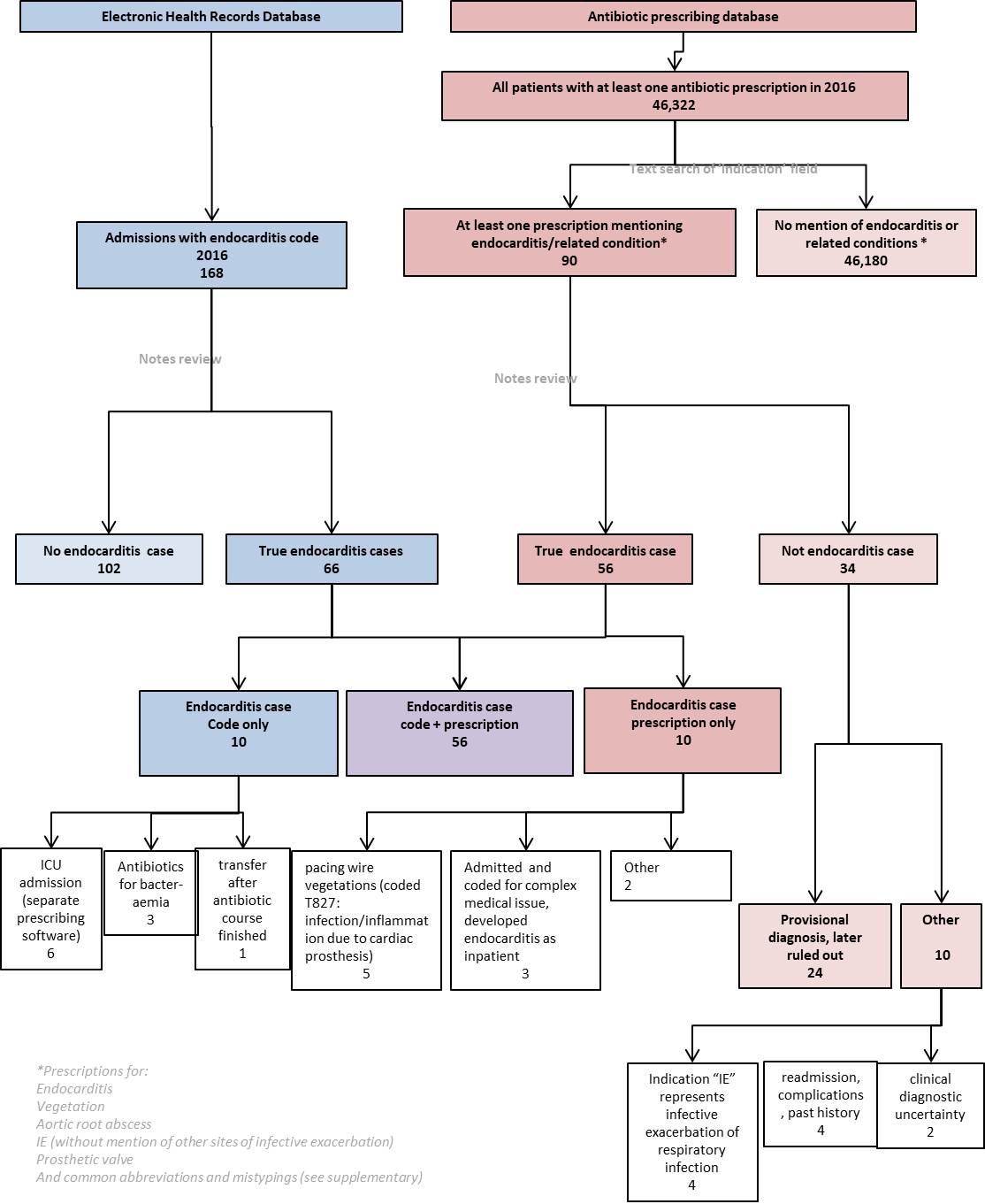


**Table S4** Reviews and Duke status in the Leeds Service Database and matching to endocarditis coded admissions

| Duke Status | Both datasets | Coding dataset only | Clinical dataset only | Total |
| --- | --- | --- | --- | --- |
| Definite | 560 | 0 | 153 | 713 |
| Possible | 178 | 0 | 110 | 288 |
| Rejected | 101 | 0 | 818 | 919 |
| --- | 0 | 842 | 0 | 842 |
| Total | 839 | 842 | 1,081 | 2,762 |

**Table S5** Agreement between different combinations of endocarditis-coded admissions and confirmed clinical cases in Oxford

|  | removal of admissions with: | | |  |  |  |  | proportion audit |  |  | proportion audit |
| --- | --- | --- | --- | --- | --- | --- | --- | --- | --- | --- | --- |
|  | <3day | readm | elective |  |  |  |  | true cases correctly |  |  | non-cases correctly |
| Codes used | LOS | <30days | status | True Positive | False Positive | True Negative | False Negative | identified | Specificity | PPV | identified |
| I33.0 primary only |  |  |  | 231 | 42 | 203 | 76 | 0.75 | 0.83 | 0.85 | 0.73 |
| I33.0 primary only | ✓ |  |  | 230 | 33 | 212 | 77 | 0.75 | 0.87 | 0.88 | 0.73 |
| I33.0 primary only | ✓ | ✓ |  | 228 | 26 | 219 | 79 | 0.74 | 0.89 | 0.90 | 0.74 |
| I33.0 primary only | ✓ | ✓ | ✓ | 223 | 23 | 222 | 84 | 0.73 | 0.91 | 0.91 | 0.73 |
|  |  |  |  |  |  |  |  |  |  |  |  |
| All primary codes |  |  |  | 253 | 79 | 166 | 54 | 0.82 | 0.68 | 0.76 | 0.76 |
| All primary codes | ✓ |  |  | 252 | 53 | 192 | 55 | 0.82 | 0.78 | 0.83 | 0.78 |
| All primary codes | ✓ | ✓ |  | 249 | 45 | 200 | 58 | 0.81 | 0.82 | 0.85 | 0.78 |
| All primary codes | ✓ | ✓ | ✓ | 243 | 38 | 207 | 64 | 0.79 | 0.85 | 0.87 | 0.76 |
|  |  |  |  |  |  |  |  |  |  |  |  |
| I33.0 in any position |  |  |  | 281 | 90 | 155 | 26 | 0.92 | 0.63 | 0.76 | 0.86 |
| I33.0 in any position | ✓ |  |  | 277 | 65 | 180 | 30 | 0.90 | 0.74 | 0.81 | 0.86 |
| I33.0 in any position | ✓ | ✓ |  | 273 | 46 | 199 | 34 | 0.89 | 0.81 | 0.86 | 0.85 |
| I33.0 in any position | ✓ | ✓ | ✓ | 265 | 39 | 206 | 42 | 0.86 | 0.84 | 0.87 | 0.83 |
|  |  |  |  |  |  |  |  |  |  |  |  |
| All primary and I33.0 secondary |  |  |  | 299 | 127 | 118 | 8 | 0.97 | 0.48 | 0.70 | 0.94 |
| All primary and I33.0 secondary | ✓ |  |  | 295 | 85 | 160 | 12 | 0.96 | 0.65 | 0.78 | 0.93 |
| All primary and I33.0 secondary | ✓ | ✓ |  | 290 | 65 | 180 | 17 | 0.95 | 0.74 | 0.82 | 0.91 |
| All primary and I33.0 secondary | ✓ | ✓ | ✓ | 281 | 54 | 191 | 26 | 0.92 | 0.78 | 0.84 | 0.88 |
|  |  |  |  |  |  |  |  |  |  |  |  |
| All codes except I38 secondary |  |  |  | 305 | 155 | 90 | 2 | 0.99 | 0.37 | 0.66 | 0.98 |
| All codes except I38 secondary | ✓ |  |  | 301 | 110 | 135 | 6 | 0.98 | 0.55 | 0.73 | 0.96 |
| All codes except I38 secondary | ✓ | ✓ |  | 294 | 89 | 156 | 13 | 0.96 | 0.64 | 0.77 | 0.92 |
| All codes except I38 secondary | ✓ | ✓ | ✓ | 284 | 69 | 176 | 23 | 0.93 | 0.72 | 0.81 | 0.88 |
|  |  |  |  |  |  |  |  |  |  |  |  |
| All codes |  |  |  | 307 | 245 | 0 | 0 | 1.00 | 0.00 | 0.56 | 0.00 |
| All codes | ✓ |  |  | 303 | 158 | 87 | 4 | 0.99 | 0.36 | 0.66 | 0.96 |
| All codes | ✓ | ✓ |  | 296 | 138 | 107 | 11 | 0.96 | 0.44 | 0.68 | 0.91 |
| All codes | ✓ | ✓ | ✓ | 286 | 115 | 130 | 21 | 0.93 | 0.53 | 0.71 | 0.86 |

Based on all admissions 2010-2016 with an endocarditis code reviewed during clinical audit (N=552). Cases of endocarditis which fulfil Duke Definite/Possible criteria counted as true cases (n=307; sum of true positive and false negative). Cases of endocarditis which do not have an endocarditis code will not be included in this dataset, thus a true measure of sensitivity and negative predictive value were not possible. Data presented as percentage of true cases found during the audit that were correctly identified by coding/administrative data combinations.

**Figure S4** Sensitivity/specificity and positive predictive values for different algorithms to identify Duke definite/possible endocarditis cases from diagnostic codes in Leeds and Oxford


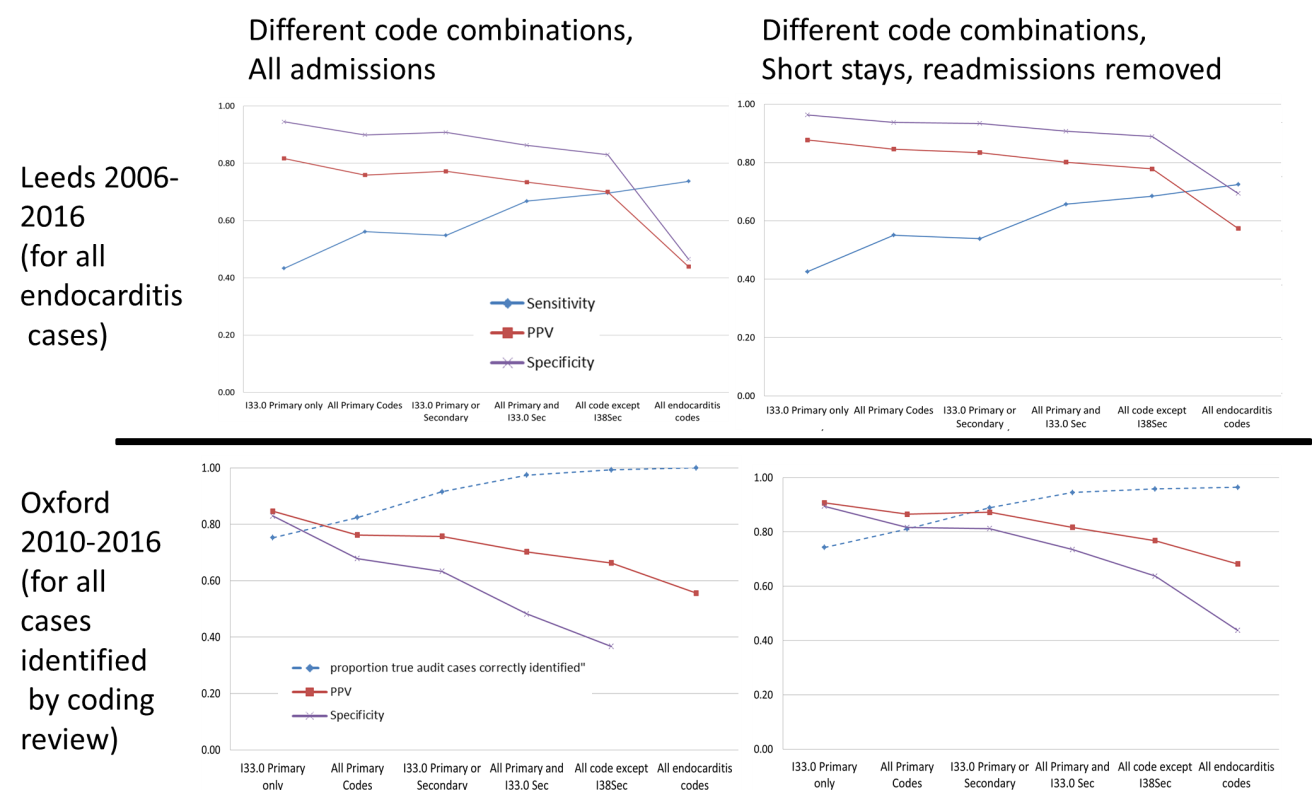


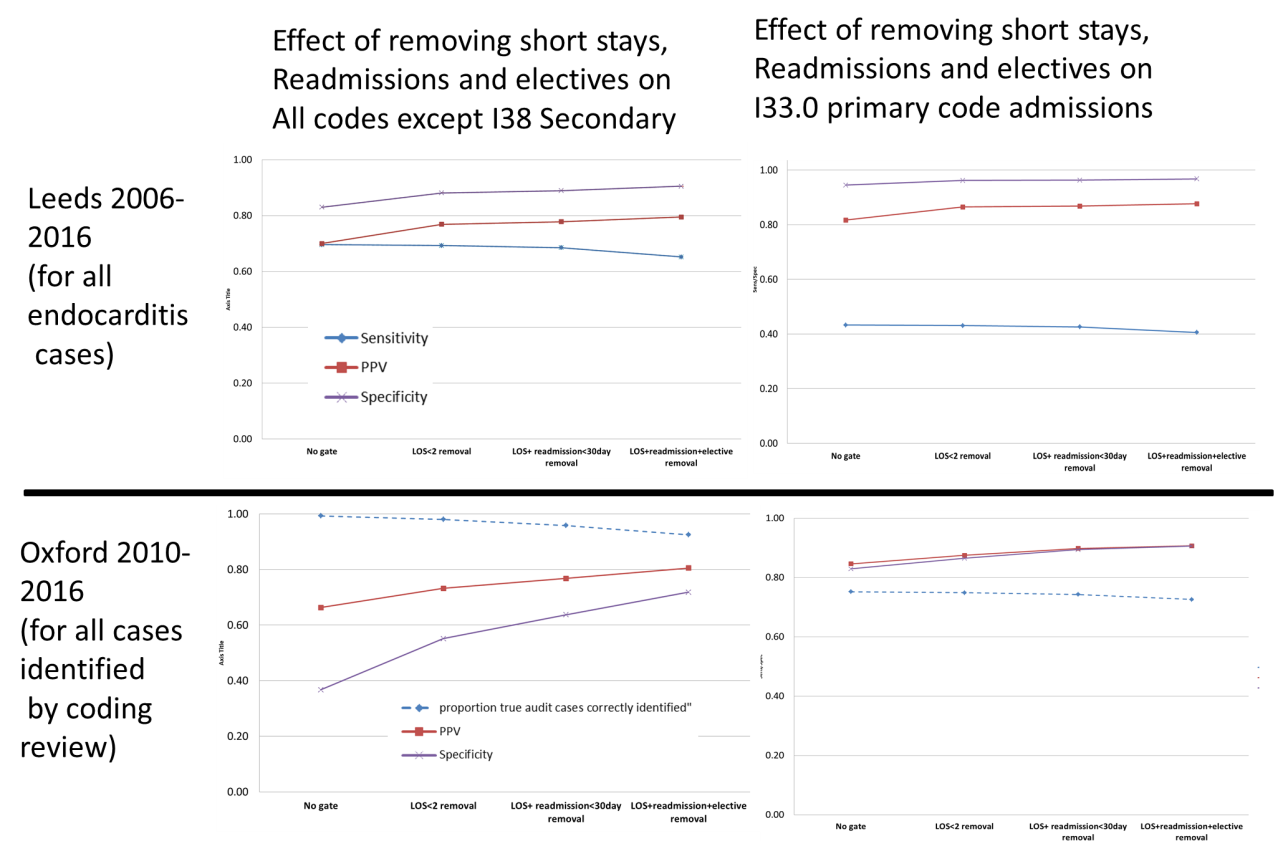


Note: Sensitivity is upper bound in Oxford providing all true cases have been identified by at least one code. Different definitions ordered by PPV.

**Figure S5** Estimated endocarditis incidence in Oxford based on diagnostic coding and administrative information


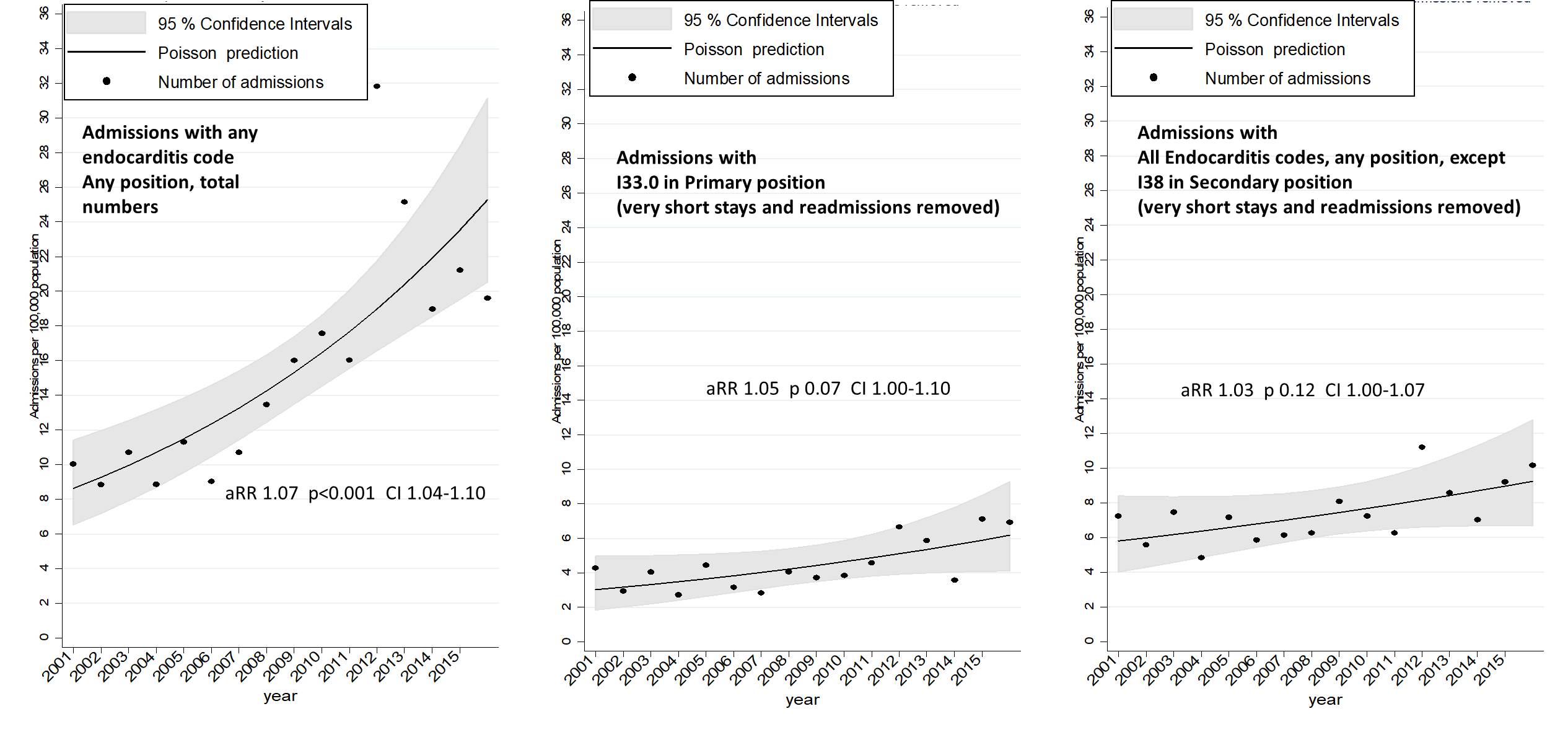


**Figure S6** Estimated endocarditis cases and causative organism from diagnostic codes compared to clinician cases, Leeds


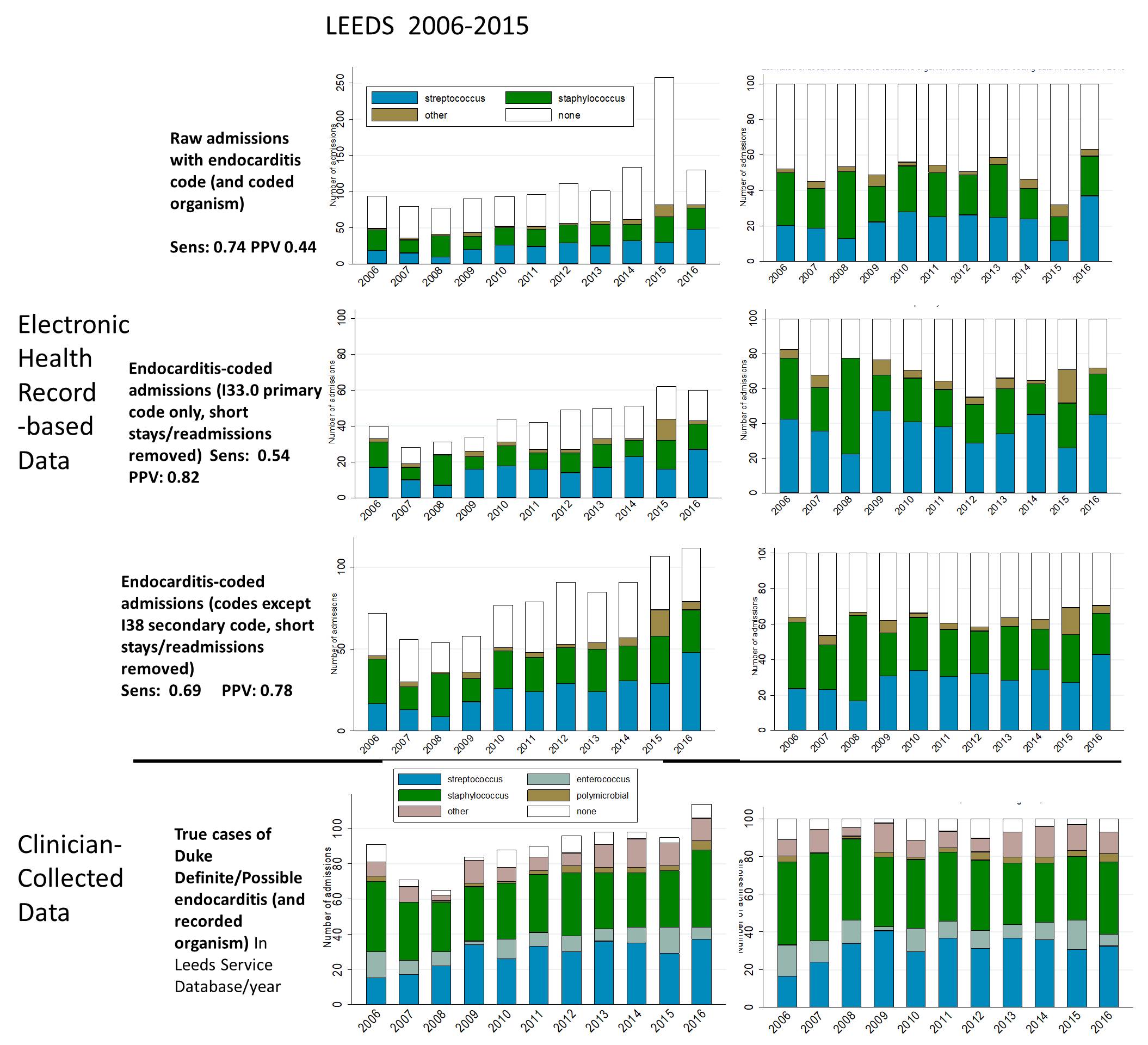


**Figure S7** Estimated endocarditis cases and causative organism from diagnostic codes and microbiological cultures, Oxford


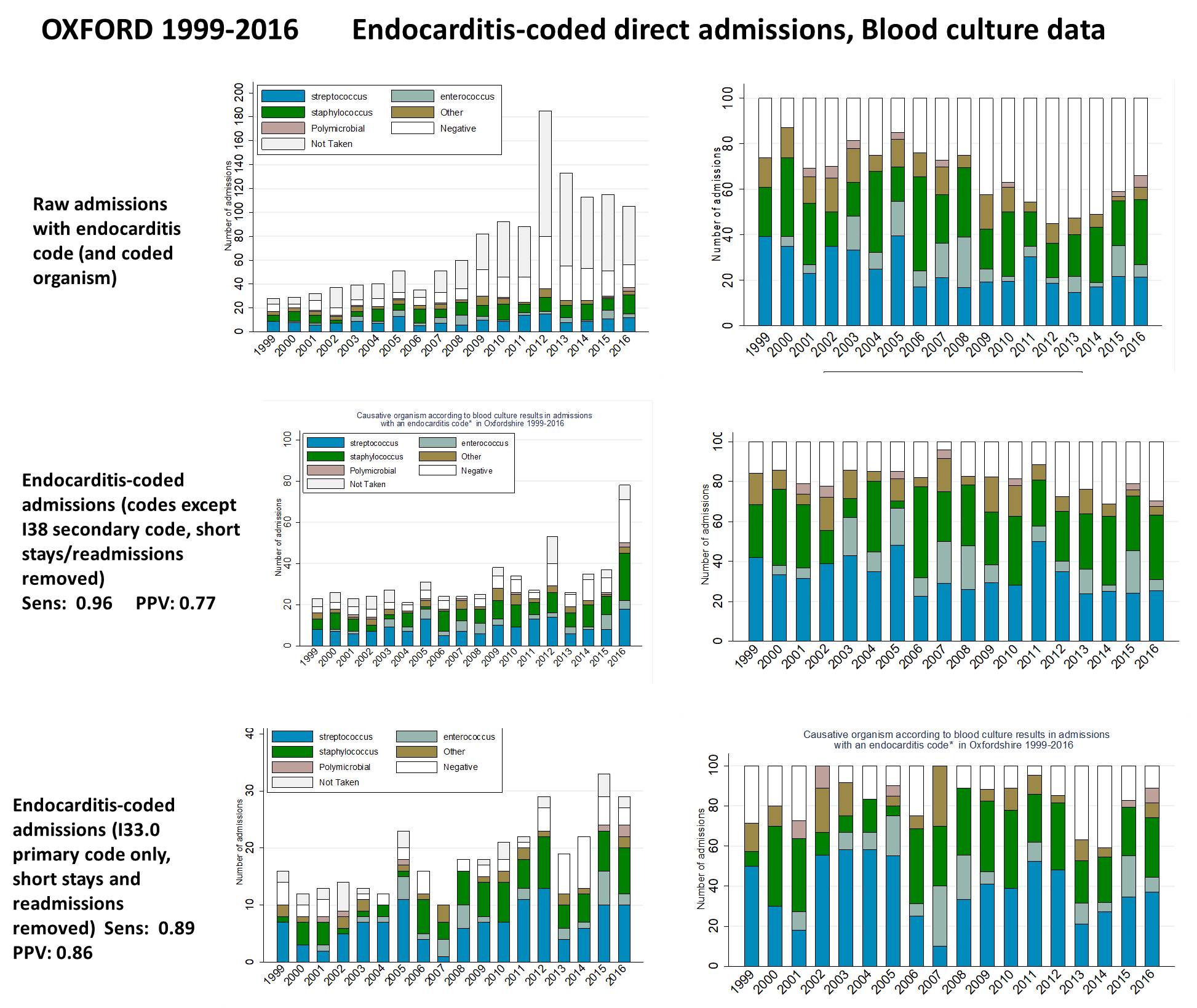


**Table S6** Coded organism vs clinician recorded organism in Leeds Duke definite/possible cases

| Leeds coded organism vs clinician recorded organism in Duke Definite/Possible cases | | | |  |  |  |  |
| --- | --- | --- | --- | --- | --- | --- | --- |
|  | clinician recorded cause (Genus) | |  |  |  |  |  |
| organism code | *Streptococcus* | *Staphylococcus* | *Enterococcus* | Other | Polymicrobial | None | Total |
| *Streptococcus* | 201 | 2 | 44 | 13 | 6* | 4 | 270 |
| *Staphylococcus* | 13 | 254 | 4 | 10 | 10* | 6 | 297 |
| Other | 6 | 0 | 16 | 23 | 1^+^ | 1 | 47 |
| None | 94 | 116 | 35 | 76 | 10^#^ | 56 | 387 |
| Total | 314 | 372 | 99 | 122 | 27 | 67 | 1001 |
| * 5 mixed *Streptococcus* and *Staphylococcus*, 1 *Enterococcus/Staphylococcus* | | | |  |  |  |  |
| ** 5 mixed *Staphylococcus* and *Streptococcus*, 1 Streptococcus and Other, | | | |  |  |  |  |
| 3 *Staphylococcus* and *Enterococcus* and 1 *Staphylococcus* and Other | | | |  |  |  |  |
| ^+^ 1 mixed *Staphyloccocus*  and Other | |  |  |  |  |  |  |
| ^#^ 4 mixed *Streptococcus* and *Staphylococcus*, 4 mixed *Staphylococcus* and other, | | | |  |  |  |  |
| 1 *Enterococcus* and Other, and 1 Other and Other | | |  |  |  |  |  |
| Note: Clinical recorded cause as recorded (genus level only). Of 614 cases where an organism code was given, 455 (74%) agreed exactly to the level of Streptococcus and Staphylococcus spp. (Enterococcus may be coded as Streptococcus given the options available). Of 475 clinician-determined Streptococcal/Staphylococcal cases, 455 (96%) were correctly identified from organism codes. | | | | | | | |

**Table S7** Coded organism vs microbiology blood culture organism in all admissions with a non-I38 endocarditis code in Oxford

|  | microbiology linked blood culture results | |  |  |  |  |  |  |  |
| --- | --- | --- | --- | --- | --- | --- | --- | --- | --- |
| coding organism | non-oral *Streptococcus spp.* | oral *Streptococcus spp.* | *Enterococcus spp.* | *Staphylococcus aureus* | *Coagulase-negative Staphylococcus spp.* | Other | Polymicrobial | none | Total |
| *Streptococcus* | 34 | 73 | 15 | 0 | 4 | 4 | 1* | 69 | 200 |
| *Staphylococcus* | 1 | 7 | 1 | 98 | 33 | 6 | 5** | 64 | 215 |
| Other | 0 | 0 | 11 | 1 | 1 | 4 | 0 | 9 | 26 |
| None | 23 | 45 | 35 | 22 | 31 | 47 | 4^#^ | 232 | 439 |
| Total | 58 | 125 | 62 | 121 | 69 | 61 | 10 | 374 | 880 |

* 1 mixed *Enterococcus* and *Staphylococus*

* 3 mixed *Staphylococcus* and *Enterococcus* ,2 mixed *Staphylococcus and* *Streptococcus*

^#^ mixed *Streptococcus* and *Enterococcus*, 3 mixed *Staphylococcus*, *Streptococcus* and Other

Note: microbiology linked results available to species level. Of 441 cases where an organism code was given, 238 (41%) agreed exactly to the level of Streptococcus and Staphylococcus spp. (Enterococcus may be coded as Streptococcus given the options available). Of 252 microbiology-determined Streptococcal/Staphylococcal cases, 238 (94%) were correctly identified from organism codes.

**Figure S8** Coding depth and use of secondary/supplementary organism codes in Leeds and Oxford


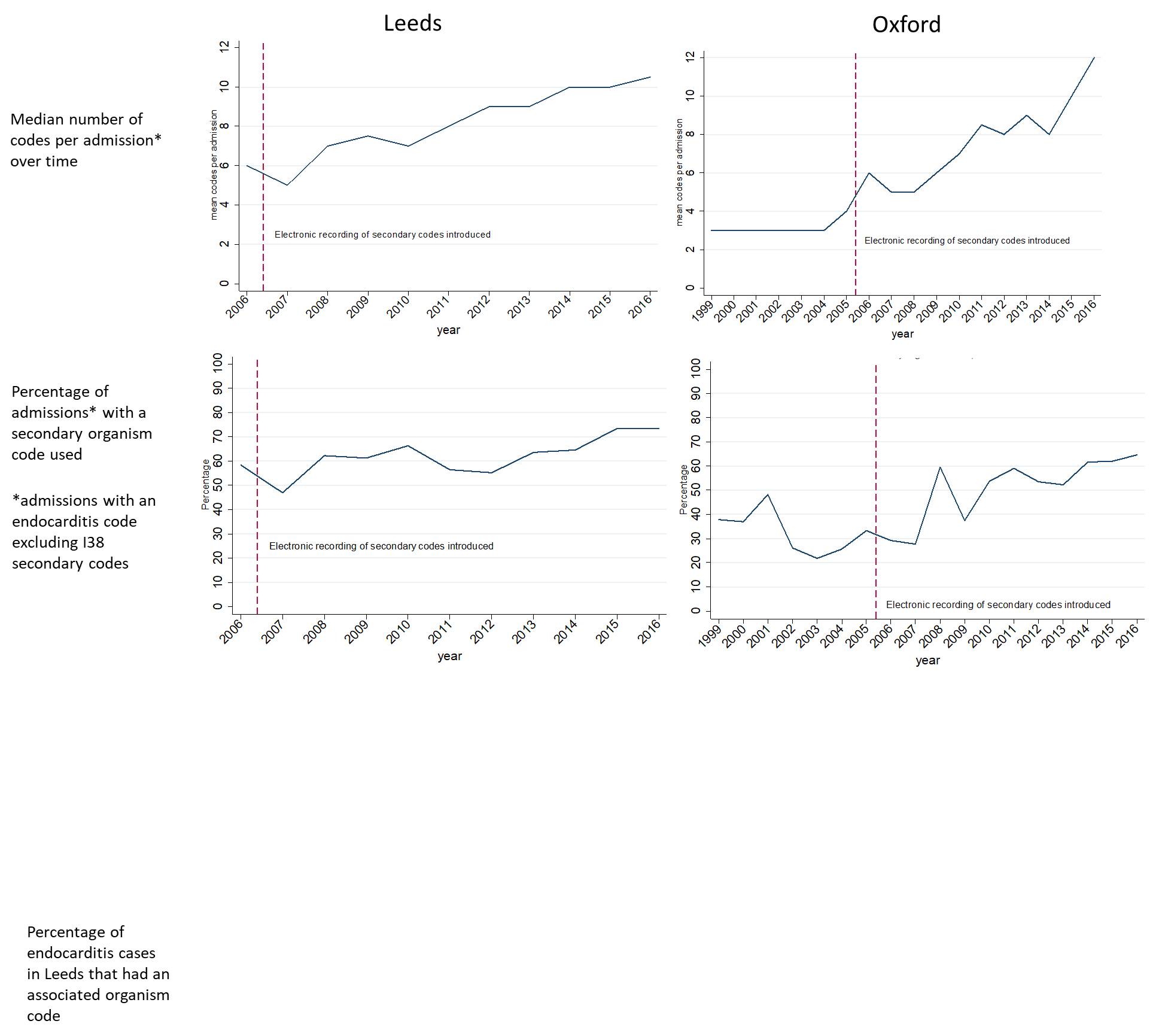

Supplement: Supplementary file 1 — Extended Methods. Table S1. Summary of studies of endocarditis incidence or features using electronic health record data or microbiological data, source of information, codes used, methods of deduplication and comparisons of codes and cases. Table S2. Summary of endocarditis codes used in the above studies. Table S3. Secondary/supplementary organism codes used and reviewed. Figure S1. Clinical reviews in the Leeds Endocarditis Service database, Duke status and diagnostic codes. Figure S2. Admissions with an endocarditis diagnosis code and selection for review: Oxford. Figure S3. Review of electronic prescription data in Oxford 2016 with matching to coded data. Table S4. Reviews and Duke status in the Leeds Service Database and matching to endocarditis-coded admissions. Table S5. Agreement between different combinations of endocarditis-coded admissions and confirmed clinical cases in Oxford. Figure S4. Sensitivity/specificity and positive predictive values for different algorithms to identify Duke definite/ possible endocarditis cases from diagnostic codes in Leeds and Oxford. Figure S5. Estimated endocarditis incidence in Oxford based on diagnostic coding and administrative information. Figure S6. Estimated endocarditis cases and causative organism from diagnostic codes compared to clinician cases, Leeds. Figure S7. Estimated endocarditis cases and causative organism from diagnostic codes and microbiological cultures, Oxford. Table S6. Coded organism vs clinician-recorded organism in Leeds Duke definite/possible cases. Table S7. Coded organism vs microbiology blood culture organism in all admissions with a non-I38 endocarditis code in Oxford. Figure S8. Coding depth and use of secondary/supplementary organism codes in Leeds and Oxford. (DOCX 2110 kb) [file 12916_2019_1390_MOESM1_ESM.docx]
